# Single-nucleus sequencing and spatial metabolomics analysis reveal the regulatory mechanism of ginkgolic acid biosynthesis in the episperm of *Ginkgo biloba*

**DOI:** 10.1093/hr/uhag064

**Published:** 2026-02-28

**Authors:** Zhi Feng, Zhi Yao, Qiye Wang, Bei Zhang, Hui Wang, Yuanqing Wang, Binlin Ai, Xingyu Zhang, Hailan Jiang, Yifan Xiao, Yiqiang Wang, Meng Li

**Affiliations:** Key Laboratory of Forestry Biotechnology of Hunan Province, Central South University of Forestry and Technology, Changsha 410004, China; Central South Academy of Inventory and Planning of National Forestry and Grassland Administration, Changsha 410014, China; Yuelushan Laboratory Carbon Sinks Forests Variety Innovation Center, Central South University of Forestry and Technology, Central South University of Forestry and Technology, Changsha 410004, China; College of Biological, Hunan Normal University, Changsha 410081, China; Central South Academy of Inventory and Planning of National Forestry and Grassland Administration, Changsha 410014, China; Yuelushan Laboratory Carbon Sinks Forests Variety Innovation Center, Central South University of Forestry and Technology, Central South University of Forestry and Technology, Changsha 410004, China; Key Laboratory of Forestry Biotechnology of Hunan Province, Central South University of Forestry and Technology, Changsha 410004, China; Institute of Tropical Bioscience and Biotechnology, Chinese Academy of Tropical Agricultural Sciences, Haikou, Hainan 571101, China; Key Laboratory of Forestry Biotechnology of Hunan Province, Central South University of Forestry and Technology, Changsha 410004, China; Yuelushan Laboratory Carbon Sinks Forests Variety Innovation Center, Central South University of Forestry and Technology, Central South University of Forestry and Technology, Changsha 410004, China; Yuelushan Laboratory Carbon Sinks Forests Variety Innovation Center, Central South University of Forestry and Technology, Central South University of Forestry and Technology, Changsha 410004, China; Key Laboratory of Forestry Biotechnology of Hunan Province, Central South University of Forestry and Technology, Changsha 410004, China; Yuelushan Laboratory Carbon Sinks Forests Variety Innovation Center, Central South University of Forestry and Technology, Central South University of Forestry and Technology, Changsha 410004, China

## Abstract

*Ginkgo biloba* is a singular and relict gymnosperm indigenous to China. Its distinctive fleshy episperm is rich in unique metabolites, ginkgolic acids, which protect the developing seed from biotic stresses. The unique nature of the tissue and its metabolites has made it highly challenging to elucidate the molecular and cellular mechanisms governing ginkgolic acid biosynthesis and regulation. In this study, we performed the mass spectrometry imaging of *G. biloba* seed, revealing that ginkgolic acids primarily accumulate in the secretory cavities of the episperm. We constructed a single-cell expression atlas of the *G. biloba* episperm and identified seven cellular types: meristem cells, subepidermal cells, lignified cells, trancheid cells, parenchymal cells, secretory cavity cells, and epidermis cells. Based on the analysis of upregulated gene expression in secretory cavity cells, pseudotime analysis of cell differentiation, and gene expression trajectory analysis, we precisely identified the key enzyme-encoding genes highly associated with ginkgolic acid biosynthesis. This approach elucidated the cellular and molecular mechanisms underlying secretory cell differentiation, secretory cavity formation, and ginkgolic acid biosynthesis and accumulation in response to exogenous jasmonic acid induction. By constructing a molecular interaction network, it was determined that the GbWRKY35, encoded by *Gb_25334*, is the core transcription factor. We further identified the signaling proteins that interact with GbWRKY35, confirming its central positive regulatory role in ginkgolic acid biosynthesis. As a core transcription factor, GbWRKY35 regulates ginkgolic acid biosynthesis through stimulating the expression of *GbAAE16*. This study provides the first spatially resolved investigation into the molecular and cellular regulatory mechanisms of ginkgolic acid biosynthesis in the episperm under jasmonic acid induction.

## Introduction

**Figure 1:**
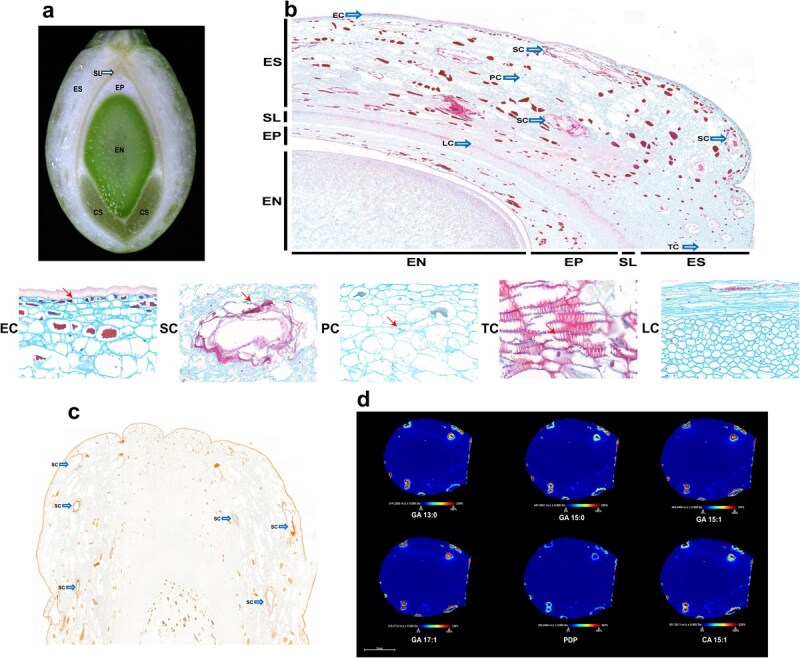
Longitudinal section image of the *G. biloba* seed. (a**)** The seed of *G. biloba* comprises five distinct structures. ES, episperm; SL, stoney layer; EP, endopleura; EN, endosperm; and CS, conoid structure. (b) The cell structures in the *G. biloba* seed at the paraffin cross section were observed. EC, epidermis cells; PC, parenchyma cells; LC, lignified cells; TC, tracheid cells; and SC, secretory cavities. The arrow denotes the corresponding cells. (c) SCs stained orange by Sudan III were observed in paraffin section. (d) MALDI images of selected ginkgolic acids ions, including GA ions (*m/z* 319.2252–*m/z* 373.2716) and long chain alkylphenol ions (*m/z* 301.2511–*m/z* 303.2665) in the *G. biloba* seed. GA 13:0: ginkgolic acid C13:0, GA 15:0: ginkgolic acid C15:0, GA 15:1: ginkgolic acid C15:1, GA 17:1: ginkgolic acid C17:1, PDP: 3-pentadecylphenol, and CA 15:1: C15:1. Different colors represent different relative contents, with darker shades of blue indicating lower content and darker shades of red indicating higher content.

*Ginkgo biloba* seeds are developmentally analogous to fruits in gymnosperms [1]. Notably, their morphology and structure resemble those of angiosperm fruits. The seed comprises four distinct tissues: the endosperm (EN), endopleura (EP), stoney layer (SL), and episperm (ES) (Fig. 1a). The EN contains a large amount of protein and starch, which are crucial for the formation of the embryo [2]. The SL is composed of sclerenchyma cells, forming a hard shell that protects the EP and the EN [3]. The EP consists of multiple layers of parenchyma cells (PCs), which are gradually expelled during seed development, eventually forming a membranous layer tightly wrapping the EN [4]. The ES of *G. biloba* seed is fleshy and contains ginkgolic acids, the unique metabolite of *G. biloba* (about 7% in dry ES), which has toxic and allergenic properties, effectively defending against insects, fungi, and bacteria [5–8]. It is a biodegradable bio-based pesticide. Additionally, as a potential medicinal material, ginkgolic acids have significant application value in antitumor and antioxidant activities [9]. Common types of ginkgolic acid (GA) include 2-hydroxy-6-tridecylbenzoic acid (GA 13:0), 2-hydroxy-6-[(8*E*)-pentadec-8-en-1-yl]benzoic acid (GA 15:1), 2-hydroxy-6-pentadecylbenzoic acid (GA 15:0), 2-(10-heptadecenyl)-6-hydroxybenzoic acid (GA 17:1), and 2-[(8*E*,11*E*)-8,11-heptadecadien-1-yl]-6-hydroxybenzoic acid (GA 17:2), with GA 15:1 being the main component, accounting for about 50% of the total ginkgolic acids [8]. There are a large number of secretory cavities (SCs) in the *G. biloba* episperm, which are speculated to be the cell tissues for accumulating lipids and phenolic ester metabolites [10]. However, due to previous technical limitations, it is still undetermined whether ginkgolic acid is precisely located in the SCs of the ES. Earlier investigations into the *G. biloba* episperm, based on transcriptome sequencing, identified acyl-CoA synthetase as the key enzyme catalyzing the acylation of C16–C18 fatty acids. Although the genes encoding these long-chain acyl-CoA synthetases (LACS) possess unusually long introns, this feature does not compromise their high expression levels. This robust gene expression is critical, as it guarantees the ample provision of necessary substrates, ultimately determining the rate of ginkgolic acid biosynthesis [11, 12]. However, these family genes expressed in the ES cells and which factors regulate them remain scientific questions to be resolved.

Currently, comprehensive analyses of numerous metabolites in *G. biloba* have been conducted using gas chromatography, liquid chromatography, capillary electrophoresis, and hyphenated methods [13]. However, these metabolites often lack spatial localization, as extraction of metabolites usually requires homogenizing the considerable amount tissue, which can be weighty and voluminous. Generally, plants contain 10 basic tissue types and a large number of structurally different cell types [14]. Plant metabolites are often synthesized and metabolized in specific cell groups rather than being uniformly distributed throughout the tissue. To determine the spatial localization and relative content of various metabolites, and to explore the cellular molecular mechanisms of metabolite synthesis and regulation, spatial metabolomics has emerged [15]. Spatial metabolomics is an emerging technology combining metabolomics and spatial distribution data. Based on matrix-assisted laser *desorption*/*ionization* mass spectrometry imaging (MALDI-MSI) technology, it acquires spatial chemical information of metabolites in different plant organs and tissues from cross-sections of plant samples. This technology has been applied to nonmodel plants such as *Fragaria × ananassa* [16], *Lycium barbarum* [17], *Salvia miltiorrhiza* [18], and *Camellia sinensis* [19]. Spatially resolved metabolite information has enhanced the understanding of metabolic characteristics in these species. The application of this technique to *G. biloba* enables a more precise characterization of the distribution patterns of specialized metabolites within its seeds.

Conventional transcriptome sequencing analysis is often conducted on multicellular and multitissue plant materials for RNA extraction, cDNA library construction, and sequencing studies. The genes in different cellular tissues of the research materials have differential expression levels, making it impossible to accurately identify the functional key genes at the cellular level for secondary metabolite biosynthesis, environmental signal transduction, and phytohormone signal transduction, which limits the advancement of research on plant-specific phenotypes and metabolic mechanisms. Single-cell sequencing technology can provide high-resolution transcriptome data at the cellular level, analyze the gene expression differences in plant cells, and thus more deeply understand the cellular heterogeneity of plant metabolic processes [20]. At the same time, it can reveal the transcriptional differences among different cells of the same tissue and, through the identification of key genes and the construction of regulatory networks, reveal the response mechanisms of plants to environmental or phytohormone signals [21]. Single-nucleus sequencing is the latest technology in single-cell sequencing. By extracting plant tissue nuclei, it provides systematic single-cell level identification of multicellular tissues for nonmodel plants that cannot effectively produce protoplasts [22]. It also analyzes the transcription factor genes and encoding-enzyme genes expression of different-type cells and displays the differentiation trajectories of different cellular clusters. This technology allows us to gain an in-depth understanding of the expression levels and regulation mechanisms of ginkgolic acid biosynthesis-related genes in the ESs. Although single-cell sequencing technology has been systematically used to identify cell populations in model plants such as *Arabidopsis thaliana* [23], *Zea mays* [24], *Solanum lycopersicum* [25], *Oryza sativa* [26], *Glycine max* [27], and *Nicotiana tabacum* [28], gymnosperms like *G. biloba* are nonmodel plants, lacking databases and marker genes, making the identification of individual cell populations challenging. Currently, only *Decaisnea insignis* [29] and *Hylocereus undatus* [30–32] have used single-cell sequencing to construct single-cell atlases of fruit peels. However, these studies mainly aimed to reveal the mechanisms of fruit senescence. There are no reports on the expression characteristics of cellular types and metabolic mechanisms in the fleshy episperm (false pericarp) of gymnosperms using single-cell sequencing. During the development of *G. biloba* seeds, whether different types of ES cells participate in different cellular processes and have different functions is worth exploring. Identifying the cellular cluster involved in ginkgolic acid biosynthesis and analyzing their regulatory mechanisms will provide a theoretical basis for regulation of ginkgolic acid accumulation and enhancing seed responses to biotic stress.

Based on previous findings from our research, the development of the ES was divided into five stages, demonstrating a strong correlation between endogenous jasmonic acid (JA), ginkgolic acid, and ginkgolic acid synthesis-related genes during development [3, 11]. The investigation of the effects of exogenous JA on ginkgolic acid accumulation in the ESs revealed that under 200 mg/l JA treatment, the ginkgolic acid content and the expression of ginkgolic acid synthesis-related genes in the JA treatment were significantly higher than those in the CK group at the stage when seed coat differentiates into three layers [11]. To elucidate the cellular and molecular mechanisms of ginkgolic acid biosynthesis, we integrated spatial metabolomics with single-nucleus sequencing of the episperm to precisely localize ginkgolic acid accumulation and generate a comprehensive cell-type atlas. This allowed us to identify the specific tissues and cell types involved in biosynthesis. Then, leveraging this cellular resolution for pseudotime and trajectory analysis enabled the reconstruction of episperm cell differentiation pathways, particularly those leading to SC formation, and the identification of key enzyme-encoding genes highly associated with ginkgolic acid biosynthesis activated during this process. Finally, a gene regulatory network from the differentially expressed genes in the target cell clusters was constructed. This analysis identified core transcription factors, culminating in the functional validation of *GbWRKY35* (*Gb_25334*) as a central regulator of ginkgolic acid biosynthesis. This multifaceted strategy provides a spatially resolved model for the regulation of this unique metabolite.

## Results

### Observation of cell morphology of *G. biloba* seed and identification of secretory cavity metabolites

The term ‘*G. biloba* seed’ refers to the ovulate strobilus after pollination. Our previous research found that 60 days postpollination, metabolites rapidly accumulate in the seed and the content of ginkgolic acid in the episperm significantly increases under the treatment with 200 mg/l exogenous JA [11]. At this stage, five distinct tissue structures are clearly observed: the episperm (ES), stoney layer (SL), endopleura (EP), endosperm (EN), and conoid structure (CS) (Fig. 1a). In paraffin sections, five cellular tissue types with distinct morphological characteristics are observed in the episperm: epidermis cells (ECs), parenchyma cells (PCs), lignified cells (LCs), tracheid cells (TCs), and SCs (Fig. 1b). LCs adjacent to the SL have thick cell walls that further thicken during development, forming a hard lignified shell. The outermost ES layer consists of closely arranged ECs. PCs beneath the ECs often have obscured or peripherally displaced nuclei. SCs of various sizes are present near some PCs and ECs, with cavity walls of closely arranged cells and lysing secretory cells releasing intracellular substances. TCs extend from the collar into the ES, SL, and EP, primarily for water transport (Fig. 1b). Substances within SCs stain orange-red with Sudan III, suggesting a lipid-rich composition (Fig. 1c).

To determine ginkgolic acids localized to SCs, we performed MALDI mass spectrometry on hematoxylin–eosin (HE)-stained frozen sections (Figs S1 and S2). We collected 3550 metabolite data and identified 1231 metabolites by matching to reference databases (Table S1). Four ginkgolic acids (GA 13:0, 15:0, 15:1, 17:1) and two long-chain alkylphenols (PDP and CA 15:1) were specifically enriched in SCs of the ES, with low levels in SL, EP, and EN (Fig. 1d, Fig. S3). SCs also contained alkaloids, fatty acyls, glycerophospholipids, steroids, and sphingolipids (Fig. S4). These results reveal for the first time the types of metabolites in the SCs of the *G. biloba* ES, which mainly consist of fatty acyls, phenolic lipids, and alkaloids. The unique metabolite of *G. biloba,* ginkgolic acids, is stored in this tissue to resist environmental biotic stress.

### Generation of a cell atlas of *G. biloba* episperm under JA treatment

**Figure 2:**
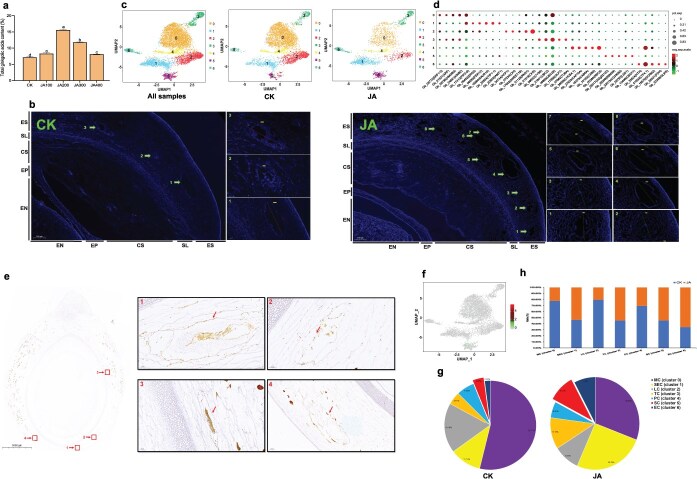
Generation of *G. biloba* episperm cell atlas and cellular cluster analysis for episperm cells under JA treatment. (a) Contents of total ginkgolic acids in the episperm under JA treatment. (b) Observation of the SCs under JA treatment in DAPI-stained paraffin sections of *G. biloba* seed. (c) UMAP visualization of seven cellular clusters in CK and JA treatment groups of *G. bilob*a episperm samples. (d) Expression patterns of representative cluster-specific marker genes on UMAP. The color scale represents the gene expression levels, with red indicating high expression and green indicating low expression. (e) RNA in *situ* hybridization of *Gb_28017*, which is a marker gene for the SC cells. The red arrows indicate cells with successful RNA *in situ* hybridization. Hematoxylin stains the cell nucleus blue, and DAB shows cell nucleus as brownish-yellow. The darker the brownish-yellow color of the cell, the higher the expression level of the marker gene. **(**f) UMAP plot of the expression distribution of 29 *GbLACS* genes in cellular clusters. (g) Pie charts depicting the percentage composition of different cellular clusters in CK and JA groups. (h) Bar charts showing the statistic proportions of CK and JA groups within each cluster of cells.

We confirmed that 200 mg/l JA (JA200) treatment most effectively induced total ginkgolic acid accumulation in the ES at 60 days postpollination, doubling the content compared to the control (CK) (Fig. 2a, Fig. S5). Therefore, JA200 (hereafter ‘JA’) was used for subsequent analyses. 4′,6-Diamidino-2-phenylindole (DAPI)-stained sections revealed JA altered morphology and number of SCs. CK had fewer, mostly mature SCs, while JA-treated ES exhibited more SCs, with many at earlier developmental stages (cells vacuolated, small spaces forming) (Fig. 2b) [33]. The SC is an important tissue for the accumulation of ginkgolic acids. We found that the content of ginkgolic acids in the ES significantly increased under JA treatment, and there was a phenomenon of further lysis and formation of new SCs. Therefore, single-nucleus sequencing technology was used to analyze the ESs on the CK group and the JA group, aiming to reveal the regulatory mechanism of ginkgolic acid biosynthesis and accumulation at the cellular and molecular level.

Nuclei from the ES were purified and used to create single-nucleus sequencing libraries via the 10× Genomics Chromium platform. Through basic quality control of the sequencing data (Table S2) and data quantification (Table S3, Fig. S6), a total of 5000 individual cells were successfully profiled in the CK group, while 3002 individual cells were successfully profiled in the JA group. After filtering out abnormal cells (Table S4, Fig. S7), 4664 cells in the CK group and 2320 cells in the JA group were obtained (Table S5). From the 8 million reads obtained from the ES samples, 91.7% and 96.6% were aligned to the *G. biloba* reference genome. Additionally, 704 median-expression genes were detected in the CK group and 358 median-expression genes were found in the JA group using the *Ginkgo* Database Genome.

The filtered cells of the CK group, the JA group, and the combined cells of both groups (All samples) were respectively subjected to cell clustering and annotation to generate a complete cell atlas of the *G. biloba* episperm. A total of 41 309 genes were identified and classified into seven distinct cellular clusters in the single-nucleus transcriptome data. Uniform Manifold Approximation and Projection (UMAP) visualization was used to display the cellular clusters in all samples, the CK group, and the JA group (Fig. 2c). The results showed that cellular cluster 0, 1, 2, 3, 4, and 6 exhibited similar relative expression abundances in both groups, whereas cellular cluster 5 had differences between the two groups.

To annotate each cellular cluster, we identified enriched cluster genes with significantly higher expression levels in specific cell clusters compared to other cell clusters (Table S6, Fig. S8). Based on the functions of these genes reported in other plant cell studies, 35 marker genes with significantly upregulated expression were screened from the seven cellular clusters for cellular cluster identification (Fig. 2d).

### Identification and analysis of cellular clusters under JA treatment

Marker gene analysis identified seven cellular clusters in *G. biloba* seeds, with functional annotations supported by RNA *in situ* hybridization and literature references (Figs S9–S22).

Cellular cluster 0 (meristematic cells, MCs) specifically expressed marker genes for primordial cells [*Gb_01521* (*ARF*, ADP-ribosylation factor) and *Gb_05796* (*HMGB3*, high mobility group B protein 1-like)] [34, 35], flowering development [*Gb_05772* (*ERF*, eukaryotic peptide chain release factor)] [36], and meristematic vasculature [*Gb_17413* (*PIP2*, plasma membrane intrinsic protein 2)] [37]. *In situ* hybridization of *Gb_05796* localized expression to the junction between the ES and collar, consistent with known meristem positions [38].

Cellular cluster 1 (subepidermal cells, SECs) specifically expressed marker genes associated with the pigment layer [*Gb_06452* (*ERG3*, elicitor-responsive protein 3)], sclerenchyma [*Gb_20903* (*IAMT1*, indole-3-acetate *O*-methyltransferase 1)], and subsidiary cells [*Gb_15664* (*SAUR10*, SAUR-like auxin-responsive protein family)] [39–41]. *In situ* hybridization of *Gb_06452* was confirmed on the inner side of epidermal cells, supporting the SEC identity [42].

Cellular cluster 2 (lignified cells, LCs) specifically expressed marker genes involved in lignin synthesis [*Gb_34744* (*XCP1*, low-temperature-induced cysteine proteinase) and *Gb_01128* (*RNS1*, ribonuclease 1)] [43, 44] and a phloem companion cell marker [*Gb_17602* (*SWEET1*, Nodulin MtN3 family protein)] [45]. *In situ* hybridization for *Gb_34744* showed expression in collar tracheids and lignified thick-walled cells.

Cellular cluster 3 (tracheid cells, TCs) specifically expressed vascular marker genes [*Gb_09532* (*CYS6*, cysteine inhibitor 1) and *Gb_17655* (*GLP7*, germin-like protein subfamily 1 member 1)] [40]. *Gb_17655* expression was localized to the ES–collar junction, an area rich in reticulated tracheids.

Cellular cluster 4 (parenchyma cells, PCs) specifically expressed multiple photosynthesis-related genes (*RBCS*, *CAB6*, *CAB8*, *CAP10*, *LHCB4*), characteristic of parenchyma [46–48]. *Gb_10163* expression was confirmed in fleshy episperm parenchyma.

Cellular cluster 6 (epidermis cells, ECs) specifically expressed known epidermal marker genes [*FDH*, 3-ketoacyl-CoA synthase 10; *SCRM2*, basic helix–loop–helix (bHLH) DNA-binding superfamily protein; *GLIP5*, GDSL esterase/lipase 5; *HTH*, glucose-methanol-choline (GMC) oxidoreductase family protein; and *LTP4*, lipid transfer protein 4] [43, 46, 48–50]. *In situ* hybridization for *Gb_05331* confirmed expression in the outermost ES layer.

Cellular cluster 5 (secretory cavity cells, SC cells) expressed trichoblast-like markers *Gb_28017* (*At3G52120*) and *Gb_05750* (*LSD1*, Snf7 domain-containing protein/zf-LSD1 domain-containing protein) [40, 51]. *In situ* hybridization of *Gb_28017* confirmed specific SC expression (Fig. 2e). GO (gene ontology) functional enrichment for this cluster included ceramide/sphingolipid metabolism (Fig. S22), consistent with spatial metabolomics finding phytosphingosine in SCs (Figs S3 and S4). Critically, 29 *GbLACS* (long-chain acyl-CoA synthase) family genes, key for ginkgolic acid biosynthesis, were predominantly expressed in SC cells (Fig. 2f, Table S8), identifying SCs as the primary site for ginkgolic acid production [11, 12].

Through the identification of cellular clusters, we determined that clusters 0, 1, 2, 3, 4, 5, and 6 are MCs, SECs, LCs, TCs, PCs, SC cells, and ECs, respectively. Using RT-qPCR to verify the expression pattern of marker genes in different cell-type tissues of the ES, the results of the verification were consistent with the identification results (Fig. S23, Table S9). Statistical analysis of cell proportions in the CK group and the JA group showed significant changes in the composition of ES cells under JA treatment. Particularly, the proportions of SC cells, SECs, and ECs increased from 3.97%, 11.13%, and 2.02% in the CK group to 9.57%, 25.78%, and 7.72% in the JA group, respectively (Fig. 2g). Conversely, the proportion of PCs decreased from 53.77% to 30.82% (Fig. 2g). Additionally, the number of SC cells, ECs, and SECs in the JA group was higher than in the CK group (Fig. 2h). The results indicated that the component composition of cellular clusters represented by SCs changed under JA treatment. The focus of subsequent analysis was to explore how the subcluster of SC cells associated with ginkgolic acid biosynthesis responded to JA signals.

### Differentiation trajectories in the development of *G. biloba* episperm cells

**Figure 3:**
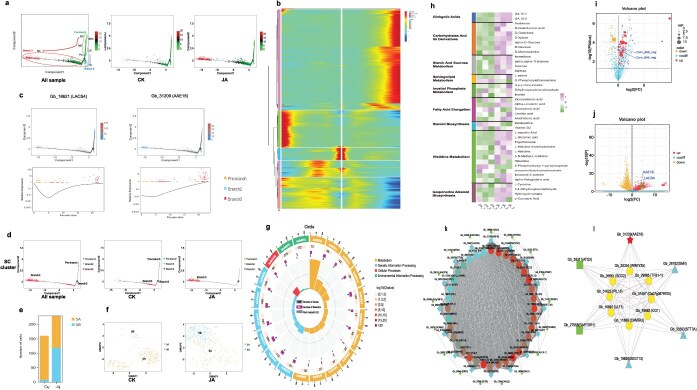
Developmental trajectories of episperm cells under JA treatment. (a) Latent time shows the internal clock of cells. Different colors of latent time represent different differentiation times, with darker shades of green indicating earlier times and darker shades of red indicating later times. (b) Clustering of differentially expressed genes along a pseudotime progression of episperm cells. (c) Visualization of the ginkgolic acid biosynthesis gene expression pattern in panel b mapped onto the pseudotime trajectory. Pseudotime mapping of each gene with expression curves below. The horizontal axis represents time progression from left to right. The figure illustrates the gene’s expression changes across three different states of cells over time. (d) RNA velocity analysis of SC cells mapped three cellular states in the pseudotime plot. The colors represent different cell states. (e) Bar charts showing the number of CK and JA sample cells within SC subcluster. (f) UMAP visualization of two cellular subclusters in CK and JA treatment groups of SC cells. (g) KEGG functional enrichment circle plot of upregulated differentially expressed genes in 5B subcluster cells. (h) Relative content of metabolites in episperms of CK and JA treatment groups, presented as cluster heatmap. (i) Volcano plot of differentially expressed metabolites in SC of two groups. (j) Volcano plot of differentially expressed genes in SC of two groups. (k) The Gene Regulatory Network (GRN) was constructed from DEGs of 51 transcription factor-encoding genes, two enzyme-encoding genes in ginkgolic acid biosynthesis, and other enzyme-encoding genes. The size of the node represents the number of gene connections. The color of the node, ranging from green to blue to gray to red, indicates the abundance of gene connectivity from low to high. Circles and diamonds represent transcription factor-encoding genes and enzyme-encoding genes, respectively. (l) Interaction network diagram of the top 8 transcription factor-encoding genes and top 6 enzyme-encoding genes with the highest connectivity scores. Yellow circles represent transcription factor-encoding genes, green rectangles represent enzyme-encoding genes in autophagy pathway, blue triangles represent enzyme-encoding genes in glycan biosynthesis, red star represents key enzyme-encoding genes highly associated with ginkgolic acid biosynthesis.

Pseudotime analysis (a method to infer cell differentiation trajectories through computational algorithms) using Monocle2 reconstructed the developmental trajectory of episperm cells. Cells from five early differentiating types (ECs, SECs, LCs, PCs, and MCs) were enriched in the prebranch state. Tracheids (TCs) were enriched in a later branch (Branch 2), while SC cells were specifically enriched in the terminal Branch 3, indicating the latest differentiation timing. This trajectory defines the SC formation path from prebranch to Branch 3 (Fig. 3a). Results were validated using the Slingshot method. Cells from JA-treated samples were more enriched in the late Branch 3 compared to controls (Fig. S24).

Differentially expressed genes (DEGs) across the trajectory were grouped into five expression clusters. Cluster 5 (pink) showed a pronounced expression increase along the trajectory toward SC formation (prebranch → Branch 3) and contained key genes for ginkgolic acid biosynthesis and SC development (Fig. 3b, Table S9). Notably, the *LACS* family genes [*Gb_18621* (*GbLACS4*) and *Gb_31209* (*GbAAE16*)], which are the main rate-limiting enzyme genes in ginkgolic acid biosynthesis [11, 12], were upregulated in this cluster during the transition to Branch 3 (Fig. 3c). This indicates that the transcriptional program for ginkgolic acid biosynthesis is activated during SC differentiation.

### Integrating single-nucleus sequencing and metabolomics reveal cellular process in secretory cavity under JA treatment

Pseudotime analysis indicated that JA treatment increased the proportion of cells differentiating into the late Branch 3 state, which is enriched for SC cells (Fig. 3a, d and Fig. S25). To reveal the intrinsic mechanism of thedifferentiation of the SC cellular cluster induced by JA, the SC cellular cluster was subjected to secondary clustering analysis, which was divided into two subclusters, 5A and 5B (Fig. 3e). The number of 5A cells in the CK and JA groups is similar, showing no significant change due to JA treatment. In contrast, the number of 5B cells is minimal in the CK group but increases under JA treatment (Fig. 3f). Therefore, the transcriptional profile of the 5B subcluster suggests it may represent a cellular state associated with active SC formation or maturation induced by exogenous JA.

Differential expression analysis between cellular subclusters 5A and 5B identified JA-induced transcriptional changes. KEGG enrichment of genes upregulated in 5B highlighted key processes (Fig. 3g and Table S10). In metabolism, this includes the biosynthesis of various glycans [N-glycan biosynthesis (Ko00510), various types of N-glycan biosynthesis (Ko00513), and other types of *O*-glycan biosynthesis (Ko00514)], carbohydrate metabolism [starch and sucrose metabolism (Ko00500), and inositol phosphate metabolism (Ko00562)], lipid metabolism [sphingolipid metabolism (Ko00600) and steroid biosynthesis (Ko00100)], amino acid metabolism [histidine metabolism (Ko00340)], and the synthesis of various other secondary metabolites [isoquinoline alkaloid biosynthesis (Ko00950)]. Cartayrade *et al.*’s microscopic observation of the SCs of *G. biloba* revealed that the formation of a glycan wall is a hallmark event in the formation of the episperm SCs. Therefore, the enrichment of glycan biosynthesis pathways in the 5B cellular subcluster is consistent with active preparation for SC formation, a process that could involve differentiation, accelerated maturation, or a pronounced shift in the transcriptional program of existing cells [10]. During the formation of SCs in *G. biloba*, the organelles of secretory cells are gradually degraded, and plastids and vacuoles continuously enlarge, subsequently leading to cell lysis, which releases a large number of metabolites and forms the cavity space. In the cellular processes, the pathway autophagy (Ko04136) is significantly enriched, indicating that cells in the 5B subcluster are undergoing autophagy, degrading the organelle, then undergoing lysis to release metabolites. In genetic information processing, pathways related to transcription such as basal transcription factors (Ko03022), spliceosome (Ko03040), and ribosome biogenesis in eukaryotes (Ko03008) are enriched. In environmental information processing, upregulated DEGs are mainly enriched in pathways like plant hormone signal transduction (Ko04075) and MAPK signaling pathway–plant (Ko04016).

Metabolomic profiling (HPLC-MS/MS) of SC tissues revealed significant JA-induced compositional changes (Fig. S27). KEGG enrichment analysis identified increased relative content of metabolites in several pathways, such as starch/sucrose metabolism and steroid biosynthesis (Fig. 3h). Notably, the ginkgolic acids GA 15:1 and GA 15:0 were among the differentially expressed metabolites (DEMs) that increased under JA treatment (Fig. 3i, Fig. S28). Correspondingly, in the JA-induced secretory cells (subcluster 5B), expression of the key biosynthetic genes *GbLACS4* and *GbAAE16* was significantly elevated (Fig. 3j).

The DEGs *Gb_18621* and *Gb_31209*, identified from SC cellular cluster and JA-induced differentiation, are homologs of *LACS4* and *AAE16*, respectively, and belong to the *LACS* family (acyl-activating enzymes I subclass; Fig. S29). Their expression strongly correlated with both SC formation and ginkgolic acid accumulation, matching the increased content of ginkgolic acids under JA treatment. Cellular and molecular-level screening thus identified *GbLACS4* and *GbAAE16* as key enzyme-encoding genes highly associated with ginkgolic acid biosynthesis in SC cells. Together, these results indicate that JA triggers autophagic lysis and cavity formation, concurrently upregulating these biosynthetic genes and promoting ginkgolic acid accumulation in the SC.

Under the JA treatment, upregulated genes of the SC cluster are enriched in glycan biosynthesis, autophagy, and ginkgolic acid biosynthesis. To explore the regulatory mechanisms of the formation of the SC and the biosynthesis of ginkgolic acid in the ES, a molecular network integrating differentially expressed transcription factors (TFs) and pathway enzymes was constructed (Fig. 3k, Tables S12 and S13). Network analysis based on connectivity scores identified a hub centered on the key ginkgolic acid biosynthesis gene *Gb_31209* (*GbAAE16*) (Fig. 3l, Table S14). Other upregulated network components included specific glycan biosynthesis [*Gb_19824* (*XEG113*), *Gb_26182* (*GMII*), *Gb_33393* (*STT3A*)] and autophagy [*Gb_39311* (*ATG2*) and *Gb_27558* (*RAPTOR1*)] genes in JA-treated SCs (Fig. S30).

Among the top-scoring TFs (transcription factors) in this network, eight were notably upregulated. Their co-expression with genes from the aforementioned pathways suggests they may coordinately regulate SC formation and ginkgolic acid synthesis. These TFs include genes with putative roles in DNA repair [*Gb_29885* (*TFB1–1*)], chromosome stability [*Gb_05661* (*SCC2*)], light/gibberellin signaling [*Gb_01625* (*PIL15*)], and plant–pathogen interaction [*Gb_25334* (*GbWRKY35*)], alongside several with unknown function (Table S15). Given JA’s established role in plant defense and the known function of WRKY transcription factors in mediating JA signals and phytoalexin production [52–54], we focused subsequent analysis on the interactions of the WRKY transcription factor Gb_25334 with promoters of key enzyme-encoding genes highly associated with ginkgolic acid biosynthesis and other signaling components.

### Identification of the core transcription factor regulating the response to JA in secretory cavity

**Figure 4:**
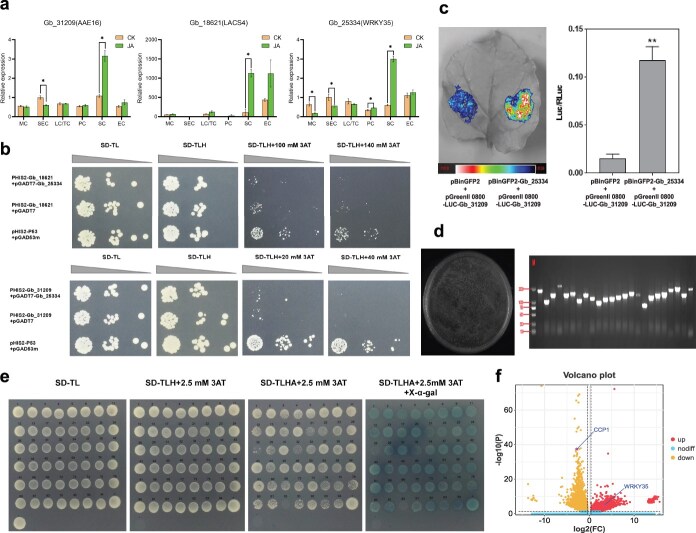
Validation of the interaction regulation of Gb_25334 (GbWRKY35). (a) qRT-PCR expression analysis of two key enzyme-encoding genes (*Gb_31209* and *Gb_18621*) highly associated with ginkgolic acid biosynthesis and the WRKY transcription factor-encoding gene *Gb_25334*. The asterisk (*) represents *P* < 0.05. (b) Interaction of the *Gb_25334* (*GbWRKY35*) with the promoters of *Gb_31209* (*GbAAE16*) and *Gb_18621* (*GbLACS4*) in yeast one-hybrid assay. pHIS2-Gb_18621*pro* + pGADT7-*Gb_25334*: experimental group, pHIS2-Gb_31209*pro* + pGADT7-*Gb_25334*: experimental group, pHIS2-Gb_18621*pro* + pGADT7: negative control group, pHIS2-Gb_31209*pro* + pGADT7: negative control group, pHIS2-p53 + pGAD53m: positive control group. (c) Interaction of the *Gb_25334* (*GbWRKY35*) with the promoter of *Gb_31209* (*GbAAE16*) in dual-luciferase assay. pBinGFP2 + pGreenII 0800-LUC-GbAAE16*pro*: control group; pBinGFP2-*GbWRKY35* + pGreenII 0800-LUC-GbAAE16*pro*: experimental group. The color ranges from blue to green to yellow to red, representing different fluorescence detection wavelengths. The asterisk symbol (**) indicates *P* < 0.01. (d) Identification of the *G. biloba* yeast hybridization library. Left: Titration identification of yeast hybridization library. Dilution factor: 100-fold, plating volume: 50 μl, and colony count: 800. Right: Identification of the insert fragment size and recombination rate of the library. (e) Yeast two-hybrid interaction screening from the *Gb_25334* and yeast two-hybrid library. Numbers 1–47 represent the monoclonal colonies that interact with the WRKY transcription factor Gb_25334. (+) is the positive control pGBKT7-*p53*+pGADT7-largeT, (−) is the negative control pGBKT7-laminC+pGADT7-largeT. (f) Volcano plot of DEGs in SC cluster under JA treatment. Expression of *Gb_25334* (*GbWRKY35*) is significantly upregulated, while expression of *Gb_13610* (*GbCCP1*) is significantly downregulated.

Utilizing RT-qPCR, we validated the expression patterns of the enzyme-encoding genes *Gb_31209* (*GbAAE16*) and *Gb_18621* (*GbLACS4*) as well as the transcription factor-encoding gene *Gb_25334* (*GbWRKY35*) involved in ginkgolic acid biosynthesis (Table S16). As shown in Fig. 4a, *Gb_18621*, *Gb_31209*, and *Gb_25334* were mainly highly expressed in the SC cells, and their expression levels in the SC cells were significantly increased under the JA treatment, which was consistent with the gene expression trend in the single-nucleus sequencing data. To confirm the regulatory function of Gb_25334 (GbWRKY35) on the expression of the two enzyme-encoding genes, we characterized it as a hydrophilic, nontransmembrane type II d WRKY factor (Figs S31–S33). Yeast one-hybrid assays revealed that GbWRKY35 interacts specifically with the promoter of *Gb_31209* (*GbAAE16*), but not *Gb_18621* (*GbLACS4*) (Fig. 4b, Figs S34 and S35). Subsequently, the dual-luciferase assay confirmed that GbWRKY35 could bind to the promoter of *Gb_31209* (*GbAAE16*) and increase the expression of luciferase (Fig. 4c). In plant pathogen interactions and phytohormone signaling pathways, WRKY transcription factors can be regulated by various signal proteins, thereby controlling the expression of target enzyme-encoding genes and the biosynthesis of metabolites. To further confirm the interaction relationships between GbWRKY35 and other proteins, we constructed a yeast two-hybrid library for *G. biloba* (Fig. 4d) and conducted yeast two-hybrid interaction assay with GbWRKY35. A total of 96 single clonal colonies were screened (Figs S36–S38). Screening by PCR amplification of positive yeast clones, Seqman and BLAST alignment, 47 *G. biloba* genes encoding proteins were found to interact with the GbWRKY35 transcription factor encoded by Gb_25334 (Fig. 4e, Table S17). Annotation analysis showed that among the 47 interacting genes, 24 genes encoded proteins with homologous protein characteristics, and 9 genes were identified to have KEGG annotation functions. Among them, *Gb_13610* encodes cysteine proteinase 1 (CCP1). As a homolog of *Arabidopsis* RD19, CCP1 is involved in plant immune response and WRKY interaction [55, 56]. In this study, it was found that under JA treatment, the expression level of *Gb_13610* (*GbCCP1*) was significantly downregulated in the SC cellular cluster, while the expression level of *Gb_25334* (*GbWRKY35*) was significantly upregulated (Fig. 4f). This suggests that JA-mediated repression of *Gb_13610* (*GbCCP1*) may release inhibition of GbWRKY35, allowing it to activate *Gb_31209* (*GbAAE16*) and promote ginkgolic acid biosynthesis.

**Figure 5:**
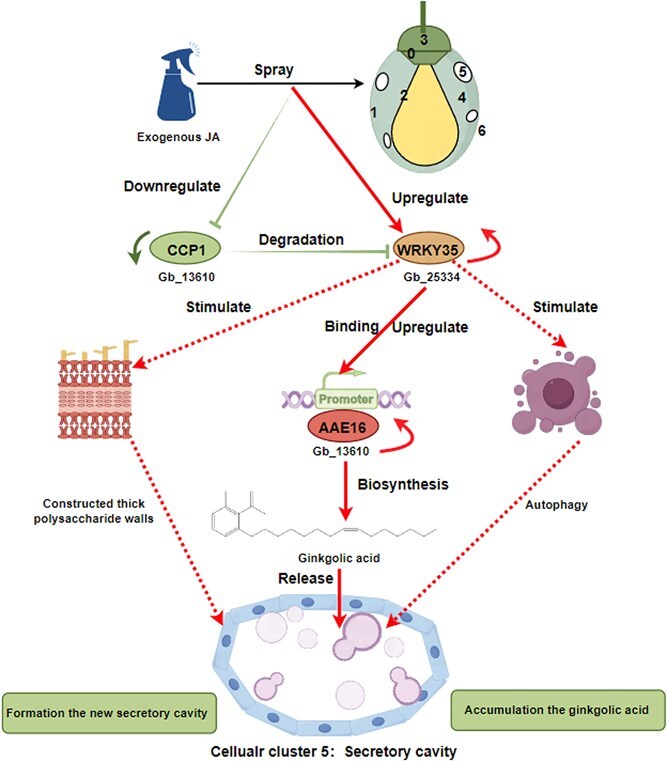
The cellular and molecular regulatory pathways of ginkgolic acid biosynthesis in the episperm of *G. biloba* seed.

In summary, our results identified the SC of episperm as an important tissue for the accumulation of ginkgolic acid, revealing the molecular mechanism of the formation of the SC and the increase in ginkgolic acid content under JA treatment. We have unearthed the core transcription factors in the regulation process and constructed a complete molecular regulation network by multiple interaction assays (Fig. 5).

## Discussion

The *G. biloba* seed coat and the pericarp of drupes share many similarities. The *G. biloba* episperm includes a layer of closely arranged epidermis cells covered with a cuticle and a layer of fleshy cells rich in hydroxypropyl cellulose, similar to the exocarp and mesocarp of drupes (Fig. S39). The lignified stoney layer and the multilayered membranous endopleura are structurally similar to the endocarp of drupes. Generally, the exocarp and mesocarp contain epidermis cells, subepidermal cells, parenchyma cells, collenchyma cells, and pigment cells. Additionally, some mesocarps are rich in vascular bundle tissues, which are involved in the transport of substances [57–60]. The *G. biloba* episperm also contains epidermis cells, parenchyma cells, lignified cells, etc., and its cell composition is similar to that of the pericarp of drupes. The *G. biloba* episperm also contains a unique secretory tissue, SCs, which have not been reported in similar studies on the pericarp and seed coat of other plants [61]. *Ginkgo biloba* contains schizo-lysigenous SCs in its stems, leaves, bud scales, and seeds, but not in its roots [62, 63]. Cartayrade extracted and detected substances from the SCs of stems and suggested that they should be rich in phenolic esters [10]. Li *et al.* [64] used MALDI-MSI technology to find small SCs at the edges of *G. biloba* leaves, containing various ginkgolic acids. However, the total ginkgolic acids content in *G. biloba* leaves is only 0.05%–0.5% [65], much lower than the total ginkgolic acids content in *G. biloba episperm* (about 6%–8%) [11]. Spatial mass spectrometry imaging of *G. biloba* seeds revealed that compared to leaf SCs, the number and size of episperm SCs are greater, and ginkgolic acids and other alkylphenols are mainly present in these SCs, scarcely detectable in the stoney layer, endopleura, endosperm, or other tissues of episperm (Fig. 2f). Therefore, we speculate that the number and size of SCs in *G. biloba* tissues determine the accumulation level of ginkgolic acids. A large number of glycerophospholipids and fatty acyl substances were also identified in episperm SCs. As volatile components in fruit peels, they might form volatile essential oils with a distinctive odor together with compounds like indole in episperm [66–68]. Additionally, steroid derivatives such as cardenolides were identified in episperm SCs (Fig. S4). Cardenolides exert toxicity by inhibiting Na+/K+-ATPase in organisms, altering membrane potentials, and causing ion imbalance within cells, inducing vomiting in mammals and birds and acting as a negative deterrent to insects [69–72]. Two alkaloids, alangimarckine and adiantifoline, were also identified mainly in episperm SCs (Fig. S4). Alkaloids have significant antipest and antidisease properties, and their bitterness and toxicity effectively prevent herbivores and insects from feeding on plants, thereby protecting the plants. Through the mass spectrometry imaging identification of *G. biloba* seed in this study, we found that episperm SCs could accumulate various toxic secondary metabolites, mainly ginkgolic acids, which collectively protect the developing seeds from biological threats.

As a gymnosperm that evolved as a single genus and species, *G. biloba* possesses a unique seed structure. Its episperm resembles the pericarp and flesh of apricots and peaches in morphology [73]. In this study, single-nucleus sequencing of the episperm identified seven cellular clusters, whereas the previously published single-cell sequencing databases for the pericarp and flesh of *D. insignis* [31] and *H. undatus* [30] identified 15 and 16 cellular clusters, respectively. The number of cellular clusters in the *G. biloba* episperm is lower than that of the pericarp and flesh of angiosperms, which may be related to its status as a gymnosperm with relatively simple tissue structure. However, the lower number of cellular clusters does not alleviate the challenge of annotating cellular cluster types, as marker gene information for gymnosperms and *G. biloba* has not yet been identified. Therefore, by utilizing marker gene information from model plants (*A. thaliana*, *O. sativa*, *N. tabacum*, etc.) [74], homologous marker genes were screened from the upregulated differentially expressed genes in each gene cluster, and RNA *in situ* hybridization was performed, successfully identifying the types of the seven cell clusters: meristem cells (cluster 0), subepidermal cells (cluster 1), lignified cells (cluster 2), tracheid cells (cluster 3), parenchyma cells (cluster 4), secretory cavity cells (cluster 5), and epidermis cells (cluster 6) (Fig. 2e, Figs S9–S21). Different from the other six cellular clusters, SC cells currently lack clear marker genes in the model plant databases. From the upregulated differentially expressed genes of cluster 5, we screened genes *Gb_28017* and *Gb_05750*, which are homologous to *At3G52120* and *LSD1* in *A. thaliana*, respectively. Both genes are annotated as trichoblast marker genes in the Plantcellmarker database (https://www.tobaccodb.org/pcmdb/search). Trichoblasts and trichomes, which are specialized hair cells derived from epidermis cells, have similar formation mechanisms and can perform secretion and transport functions [75, 76]. Previous studies have shown that plant SCs are derived from the differentiation of epidermis cells and its surrounding cells [77, 78]. The RNA *in situ* hybridization results of the *Gb_28017* indicate its expression in the SC, suggesting that a universal marker gene may be used for the identification of secretory cells. The upregulated genes in the SC cellular cluster are enriched in pathway of ceramide metabolism and sphingolipid metabolism. Spatial mass spectrometry imaging show that the distribution areas of phytosphingosine and ginkgolic acid are consistent (Fig. S3), both accumulating in the SCs. Phytosphingosine is a naturally occurring bioactive ceramide that plays a significant role as a signaling molecule in plants, particularly in stomatal closure, programmed cell death, and defense against pathogen attacks [79–81]. HE discovered that the efficient expression of the *LACS* gene in *G. biloba* affected the biosynthesis efficiency of ginkgolic acid, and the expression analysis of 29 *GbLACS* genes in the single-nucleus database indicated that these genes were mainly highly expressed in the SC cellular cluster [12]. Therefore, based on the functional enrichment analysis of the upregulated genes in the SC cellular cluster, the identification of metabolites in the SC tissue, and the expression analysis of *GbLACS* genes in the cell atlas, it can be determined that the SC cellular cluster is the key cellular tissue for the biosynthesis and accumulation of ginkgolic acid.

In single-nucleus sequencing studies, pseudotime analysis models and infers possible cell development trajectories by constructing a pseudotime axis of cell differentiation, thereby obtaining the differentiation origin of target cell clusters and revealing the population changes and gene expression changes of cellular clusters induced by exogenous factors, and constructing the molecular regulatory network of key genes in the cellular differentiation process. The seed structures of gymnosperms *Cycas* and *Ginkgo* are similar, and the three seed coat layers (episperm, stony layer, and endopleura) are all developed and differentiated from a single layer of integument [73]. In the initial stage of seed development in *Cycas* and *Ginkgo*, the surface layer of the integument is composed of epidermis cells forming a cuticle to protect the tissue [82]. Subsequently, a large number of meristematic tissues beneath the cuticle undergo cell division and differentiation to promote the growth of the integument, and vascular tissues and specialized idioblast are formed during the growth of the integument, eventually enveloping the nucellus and leaving a small opening at the top of the nucellus, the micropyle [38, 73]. Therefore, the pseudotime differentiation sequence of episperm cells in this study is consistent with previous research. Epidermis cells, subepidermal cells, meristematic cells, parenchyma cells, and lignified cells are distributed in the prebranch, which are earlier differentiating cell clusters. Tracheid cell clusters gradually form dead cells during the development and maturation of the seed coat to function in water and inorganic salt transport, and thus their differentiation time is later than that of the prebranch cellular clusters. The specialized SC cells, on the other hand, are continuously formed during the development of the episperm and are located in the latest branch (Branch 3) of the entire cellular cluster differentiation timeline.

The stages of secretion cavity formation in *G. biloba* can be divided into the initial secretory cells, cells vacuolated, small spaces appeared between the cells, secretory cells dissolved, and secretory cells mature [33]. During the formation process, a large amount of autophagy and lysis occurs in the cells, a large number of lipophilic substances are synthesized in the organelles and endoplasmic reticulum of the cells, and the endoplasmic reticulum transports the secretions to the cell membrane through its transport function on the plasma membrane, mitochondria, and Golgi body, rupturing the cell membrane to release the secretions into the secretion cavity [62, 83]. At the same time, a thick layer of glycan was formed on the episperm secretion cavity [10]. Combined with cell microscopic observation (Fig. 2d), cell count statistical analysis (Fig. 2h and g), and DEGs KEGG enrichment analysis of secretion cavity cluster (Fig. 3g and h), we found that JA treatment led to an increase in the number of secretion cavities and secretory cells. Additionally, a significant upregulation of genes associated with glycan biosynthesis and autophagy was noted. The data also highlighted genetic information processes, including protein processing in the endoplasmic reticulum, ubiquitin-mediated proteolysis, ribosome biogenesis in eukaryotes, and other genetic information processing pathways. Therefore, our data support a model in which JA treatment promotes processes leading to an increased number of SC cells in the episperm. This is evidenced by the upregulation of genes associated with glycan biosynthesis and autophagy, which are crucial for cavity formation. While the increase in SC cell proportion could result from JA-induced de novo differentiation, it is also plausible that JA accelerates the maturation of preexisting precursor cells or alters their transcriptional state to execute the secretory program. Regardless of the precise cellular origin, the net effect is the acceleration of the lysis process to form more SCs, thereby promoting the biosynthesis and accumulation of ginkgolic acid. There are also reports of JA inducing the formation of secretory tissues in other plants. In *A. thaliana,* it was found that the increase in JA content can promote the formation of secretory tissue trichomes and resist biotic stresses by secreting more secondary metabolites [84, 85]. In the young stem of the Brazilian rubber tree (*Hevea brasiliensis*) treated with exogenous JA, the number of secretory tissue laticifers can be significantly increased to promote the accumulation and secretion of natural rubber [86]. JA, an important phytohormone signal responding to stress and regulating plant immunity and defense, promotes the formation of secretory tissues in various plants, promotes the biosynthesis and accumulation of metabolites, and achieves the protection function.

Spatial metabolomics and quasi-target metabolomics results indicate that ginkgolic acids primarily accumulate in the SCs, and the content of ginkgolic acids in these cavities significantly increases upon exogenous JA stimulation (Figs 1e and 3i). Ginkgolic acids, as long-chain alkyl salicylic acid derivatives, are synthesized from saturated and monounsaturated C16 and C18 fatty acids through thioesterification, condensation, reduction, dehydration, and cyclization reactions [11]. Acyl-activating enzymes (AAEs) can widely recognize the substrate and synthesize it into the corresponding CoA thioesters, while LACS enzymes, as members of the AAE family, mainly catalyze the CoA acylation of long-chain fatty acids such as palmitic acid, palmitoleic acid, and oleic acid [17, 87]. He *et al.* found that the stable high expression of the *GbLACS* gene family promoted the accumulation of ginkgolic acid [12]. This study found that the *G. biloba* LACS genes are primarily expressed in SC cellular cluster, ensuring the accumulation of ginkgolic acids (Fig. 2f). During the differentiation of other cells in the *G. biloba* episperm into SC cells and the formation of SCs under JA induction, the expression levels of two *GbLACS* genes, *Gb_18621* (*GbLACS4*) and *Gb_31209* (*GbAAE16*), significantly increased, which was highly consistent with the content changes of ginkgolic acid in the SCs (Fig. 3c and j). Therefore, based on precise analysis at the cellular and molecular levels, *Gb_18621* (*GbLACS4*) and *Gb_31209* (*GbAAE16*) were identified as key enzyme-encoding genes highly associated with ginkgolic acid biosynthesis in SC cells. Their expression levels significantly increased during the differentiation of SCs and under JA induction, promoting the biosynthesis of ginkgolic acid and increasing the content of ginkgolic acid in the SCs.

Transcription factors are vital signaling factors that receive external signals and regulate the expression level of enzyme-encoding genes to alter metabolic processes or phenotypic traits [88]. Through the construction and analysis of molecular regulatory network, we have identified eight transcription factor-encoding genes that may simultaneously regulate glycan biosynthesis, autophagy, and ginkgolic acid biosynthesis (Fig. 3l). Among these eight transcription factors, four genes (*Gb_25334*, *Gb_01625*, *Gb_18683,* and *Gb_29885*) have distinct metabolic regulatory functions. *Gb_18683* (*CG1*) and *Gb_29885* (*TFB1*) primarily control transcription and translation processes, not directly involving in signal transduction and regulation [89, 90]. *Gb_25334* and *Gb_01625* are homologous transcription factor-encoding genes of *WRKY35* and *PIL15*, respectively, mainly involved in the transduction of environmental signals. PIL15 primarily responds to gibberellin signals, promoting stem growth and inducing seed germination by inducing target gene expression [91, 92]. WRKY transcription factors primarily participate in plant–pathogen interaction pathways. Under the induction of pathogen proteins or similar signals, the expression level and enzyme activity of WRKY transcription factor expression are altered, thereby influencing the expression of defense-related genes and the accumulation of phytoalexin involved in plant protection [54, 56, 93]. The formation of the episperm secretion cavities and the accumulation of ginkgolic acid (a unique phytoalexin of *G. biloba*) are important traits for *G. biloba* seeds to exert defensive immunity and resist biotic stress. Therefore, we believe that the core transcription factor Gb_25334 (GbWRKY35) plays a crucial regulatory role in the formation of secretion cavities and the accumulation of ginkgolic acid. The formation of secretion cavities mainly involves two hallmark events: glycan biosynthesis to form thick walls of glycan and cell autophagy lysis.

Our hypothesis that GbWRKY35 may regulate glycan biosynthesis for secretory cavity walls is well supported by precedent. In *Polygonatum cyrtonema*, PcWRKY31 and PcWRKY34 can synergistically upregulate the biosynthesis of polysaccharides [94]. Utilizing whole-genome identification and analysis, it was found that nine WRKY transcription factors in *Platostoma palustre* (Blume) A.J. Paton are associated with polysaccharide biosynthesis. Their induced expression can promote glycan biosynthesis [95]. In *Cassava*, *MeWRKY20* is an upstream regulatory gene of the autophagy gene *MeATG8a*. Overexpression of *MeWRKY20* can upregulate the transcription level of ATG8a, promoting the autophagy process [96]. Similar transcriptional regulation mechanisms of WRKY transcription factors for autophagy gene *ATG* have also been verified in *Solanum tuberosum* [97, 98]. Due to the characteristic WRKYGQK sequence, WRKY transcription factors show high conservatism among different species. In this study, we believe that the selected GbWRKY35 transcription factor may effectively regulate the synthesis of polysaccharides and autophagy to achieve the goal of cell differentiation and secretion cavity formation. However, the above discussion is merely a reasonable assumption. In the future, ChIP-seq or plant-based validation should be conducted to determine the direct binding sites of GbWRKY35 on the promoters of autophagy and polysaccharide synthesis-related genes.

Although WRKY transcription factors widely participate in plant metabolic regulation, there are no reports about WRKY regulating the transcription of *LACS* and *AAE* genes. This study found that the transcription factor-encoding gene *Gb_25334* (*GbWRKY35*) and the key enzyme-encoding genes highly associated with ginkgolic acid biosynthesis, *Gb_18621* (*GbLACS4*) and *Gb_31209* (*GbAAE16*), have similar expression patterns in different cellular tissue under JA induction. Therefore, using yeast one-hybrid technology and dual-luciferase technology, our *in vitro* assays suggested that GbWRKY35 could bind with the promoter of *Gb_31209* (*GbAAE16*) (a member of the *G. biloba LACS* family) and significantly upregulate its expression, which is consistent with a role in promoting the biosynthesis of ginkgolic acid (Fig. 4b and c). It is important to note that these interaction assays, while informative, provide indirect evidence for the regulatory relationship. Direct functional validation *in planta*, such as through genetic manipulation of GbWRKY35, is currently not feasible in *G. biloba* due to the lack of an established transformation system. This technical limitation necessitates caution in interpreting the causal strength of this regulatory link. The MAPK cascade transduction is a key signaling molecule in plant resistance to fungi and pests, controlling downstream transcription factor activity through protein phosphorylation. The WRKY transcription factor, as a potential substrate for a series of MAPKs, is a key signal role of MAPK cascade transduction of plant immunity and defense [99, 100]. However, our research did not find an interaction between GbWRKY35 and related MAPK proteins in the two-hybrid screening assay of the *G. biloba* cDNA yeast library (Fig. 4 and Table S16), but found an interaction with *Gb_13610*-encoded GbCCP1 hydrolase protein. *Gb_13610* (K01373) was annotated in the plant–pathogen interaction pathway, and its encoded cysteine protease could be interacted with WRKY transcription factors and affects the expression of plant defense genes in response to external environmental stimuli. The WRKY52 transcription factor in *A. thaliana*, which resists *Ralstonia solanacearum*, has not been reported to be regulated by MAPK. When *A. thaliana* is infected with *R. solanacearum*, the bacterium’s encoded effector protein (PopP2) activates a cysteine proteinase (CCP), which further affects the enzymatic activity of WRKY52 to regulate plant immune response and defense [56, 101]. This study found that under exogenous JA treatment, the gene *Gb_13610* (*GbCCP1*) with negative regulatory function was significantly downregulated in the SC cellular clusters, while the core transcription factor-encoding gene *Gb_25334* (*GbWRKY35*) was significantly upregulated. Ultimately, as a core transcription factor, GbWRKY35 regulates ginkgolic acid biosynthesis by stimulating the expression of *GbAAE16*.in the SCs (Fig. 5).

In summary, this study for the first time completed the spatial metabolic imaging and cellular altas mapping of *G. biloba* episperm and identified the SC in the episperm as an important tissue for the biosynthesis and accumulation of ginkgolic acids. It revealed the cellular and molecular regulatory mechanism of SC formation and ginkgolic acid biosynthesis under exogenous JA induction. Specifically, by exploring the interaction between GbWRKY35 and the promoters of key enzyme-encoding genes highly associated with ginkgolic acid biosynthesis as well as other encoded proteins of *G. biloba* genes, a cellular and molecular regulatory network was constructed. Our data, primarily from correlative expression analyses and *in vitro* interaction assays, support a model in which GbWRKY35 acts as a central transcriptional regulator in this process. However, the definitive establishment of its causal role *in vivo* awaits the future development of genetic tools for *G. biloba*. The results of this study contribute to our understanding of the formation and regulatory mechanisms of the *G. biloba* episperm and its unique metabolites, and provide a theoretical foundation for the cultivation techniques of *G. bilob*a seeds aimed at controlling ginkgolic acids content.

## Materials and methods

### Preparation of *G. biloba* seed of CK group and JA group

Seeds of the *G. biloba* variety ‘Zhongnanlin No. 2’ were collected at 60 days post?pollination (the stage at which the seed coat differentiates into three layers). Samples sprayed with 0.1% polyethylene glycol 600 as CK group samples were harvested from the natural experimental base of Central South University of Forestry and Technology in Changsha, Hunan Province (latitude 28°13’ N, longitude 112°99′ E, altitude 100 m). Different concentrations of JA working solutions containing 0.1% polyethylene glycol 600 [476 μmol/l (100 mg/l), 951 μmol/l (200 mg/l), 1427 μmol/l (300 mg/l), and 1904 μmol/l (400 mg/l)] were prepared and sprayed on *G. biloba* seed. The seeds of the ‘Zhongnanlin No. 2’ *G. biloba* variety at 60 days postpollination treated with JA were collected as the JA treatment group samples. Some of the collected seeds were fixed in formalin-aceto-alcohol (FAA) solution (50% ethanol) for subsequent tissue section preparation and observation. The ESs were quickly separated and promptly cryogenically preserved in liquid nitrogen for ginkgolic acid determination, mass spectrometry spatial imaging, single-nucleus sequencing, and quasi-targeted metabolome determination. The requirements for phytohormone spraying, treatment frequency, and the quality and quantity of seed collection were referred to our previous study [11].

### Observation on the *G. biloba* seed and episperm

After 60 days of pollination, 15 *G. biloba* seeds with consistent developmental morphology were collected from the CK group for optical observation of seed longitudinal sections and paraffin section observation. The seeds fixed in FAA solution were embedded in paraffin and sectioned, then stained with safranin O and fast green, Sudan III, as well as DAPI for histological examination under a stereomicroscope (Olympus BX50F-3). The detailed procedures for section preparation refer to our previous study [3].

### *Ginkgo biloba* seed preparation for MALDI imaging

The preparation of sections, matrix spraying, mass spectrometry detection, and imaging were carried out using the device of Gene Denovo Biotechnology Co., Ltd (Guangzhou, China), and were conducted in accordance with the guidelines of Tims-tof MALDI 2. Embed the fresh *G. biloba* seeds of the CK group in a 10% gelatin (wt/vol) solution. First, preserve the tissue in a Tissue-Tek ccrymold and store at −80°C refrigerator. Equilibrate the seeds stored in a − 20°C refrigerator for 1 h, and prepare longitudinal sections of seeds with a thickness of ~15 μm using a Leica CM 1950 cryostat (Leica, Germany) following the manufacturer’s instructions. Transfer the tissue sections onto precooled ITO coated slides using a precooled brush, then dry for 20 min. Stain the sections with hematoxylin–eosin (HE) before imaging and observe the stained sections to identify the morphological structures of cells and tissues.

After labeling the sample information on the ITO tissue section slides, turn on the cyclic sprayer matrix spraying device (SunCollect, SunChrome) to perform matrix spraying. The matrix is 5 mg/ml α-cyano-4-hydroxycinnamic acid (CHCA) in a solvent of acetonitrile/0.1% formic acid (97:3, v/v) solution. The nozzle temperature is set to room temperature, with a spraying speed of 800 mm/min. A total of 12 passes are applied, with the flow rate for each layer varying between 15 and 50 μl/min.

### Mass spectrometry imaging and data analysis

Place the tissue sections with the sprayed matrix on the mass spectrometer target plate, select the intended imaging area via computer, and define the imaging resolution for 50 μm. The image is divided into a two-dimensional array of points based on its size, and detection is performed using a mass spectrometry imaging system. Mass spectrometry conditions: Thermo Q Exactive Plus mass spectrometer (Thermo Fisher Scientific, USA) coupled with an AP/MALDI (ng) UHR ion source (MassTech, Columbia, MD). The laser energy is set between 10% and 30%, and in positive ion mode, a full MS is performed first, followed by MS/MS acquisition in Data Dependent Acquisition (DDA) mode. The MS are scanned at a resolution of 35 000 over an m/z range of 81–1000, with a maximum ion injection time (MIT) set to 200 ms and an automatic gain control (AGC) of 3e6. The capillary temperature is set to 320°C, and the S-lens RF level is set to 50%. High-energy collision induced dissociation (HCD) is used for MS/MS, with the resolution of 17 500, MIT set to 50 ms, AGC set to 1e6, an isolation window of m/z 2, and a collision energy of 30 eV. During DDA analysis, top 5 ions were selected for MS/MS analysis with a dynamic exclusion [102, 103].

In mass spectrometry imaging experiments, samples on the slides are scanned row by row. Under the excitation of the laser beam, the released molecules are identified by the mass spectrometer, thereby obtaining the m/z information and peak intensity raw data for each point on the sample. The raw data are imported into SCiLS Lab software for reading, smoothing, and TIC normalization to obtain the m/z information for each point, which is then converted into pixel points on the imaging heatmap. The colored spots in the image represent the localization of compounds, with blue spots indicating low abundance at that point, and red spots indicating high abundance of the substance at that point.

### Determination of total ginkgolic acids content in *G. biloba* episperms of different groups

The CK and JA treatment groups were separated to obtain the ES. The ES of each sample was freeze-dried under vacuum. Using 1 g of dry powder as material, ultrasonic extraction was performed in petroleum ether. The extraction solvent was concentrated and redissolved in methyl ethanol solution. Additionally, the total ginkgolic acids standard sample (Shanghai Yuanye Bio-Technology Co., Ltd) was dissolved in methanol. The content determination of the standard and sample methyl ethanol solutions was performed using reversed phase high-performance liquid chromatography (RP-HPLC) on an LC-15C high-performance liquid chromatograph (Shimadzu, Japan). The detailed steps of total ginkgolic acids extraction, reagents and parameters for high-performance liquid chromatography detection, and the calculation method for the total ginkgolic acids content in the ES refer to our previous research [3].

### Library construction and single-nucleus sequencing

The fresh episperm tissues of 1.5 g each were used for nuclear extraction in both the CK group and the JA group. Five episperm tissues at the same developmental stage from each group were equally mixed. Sample preparation, nuclear isolation, library preparation, and sequencing were conducted using the devices from Gene Denovo Biotechnology Co., Ltd (Guangzhou, China), in accordance with the guidelines provided by 10× Genomics (10× Genomics, Pleasanton, CA, USA). Briefly, we collected the *G. biloba* ES from the CK group and the JA treatment group (treated with 200 mg/l JA), quickly froze them in liquid nitrogen, and then ground them into powder. The sample powder was transferred to Nuclei Isolation Buffer (NIB), and nuclear suspension was obtained after multiple rounds of centrifugation and sieving. DAPI staining solution was added to the nuclear suspension and the concentration was adjusted. Nuclei quality (DAPI signal and nuclear size) was checked using the DAPI fluorescence channel, and the nucleus was sorted and counted. The collected nuclei were diluted to ~1000 nucleus/μl [104]. Gel beads with barcode information were combined with nucleus and enzyme mixtures to form GelBeads-In Emulsions (GEMs). Subsequently, the gel beads lysed to release barcode sequences, which were reverse-transcribed into cDNA fragments and added the label to samples. The gel beads were broken, oil droplets were disrupted, and cDNA was used as a template for PCR amplification. The products of all GEMs were pooled to construct standard sequencing libraries. First, the cDNA was enzymatically fragmented into fragments of ~200 to 300 bp, and sequencing adapters P5 and sequencing primer R1 were added. Finally, PCR amplification was performed to obtain the DNA library. The constructed library was subjected to high-throughput sequencing using the paired-end sequencing mode of the Illumina sequencing platform.

### Single-nucleus sequencing data processing

The raw data from Single-nucleus sequencing underwent quality control and was aligned using the 10× Genomics Cell Ranger software (version 3.1.0) and the *G. biloba* genome (https://ginkgo.zju.edu.cn/genome/). Subsequently, barcodes and Unique Molecular Identifiers (UMIs) were filtered. Each gene ID corresponding to each barcode’s UMI was then deduplicated, and the number of unique UMIs was calculated as the expression level of that gene in that cell. Let the expected number of cells be N. Barcodes were sorted by UMI count in descending order, and the top N barcodes were selected. The 99th percentile UMI count of these barcodes was denoted as m, and barcodes with UMI count <m*10% were filtered out. The remaining barcodes were considered valid cells and further validated by Cell Ranger using the EmptyDrops method to identify low RNA content cells [105].

The R package Seurat was used for further quality control and analysis of single-nucleus transcriptome data to retain high-quality cells [106]. First, Doublet Finder (V2.0.3) was used to calculate the probability of GEMs being doublets [proportion of artificial k-nearest neighbors (pANN) value], and then the doublet rate for each sample was calculated based on the relationship between the number of valid cells (postcell ranger filtering) and the doublet rate provided by 10×. Each sample’s doublet filtering threshold was determined, and doublet filtering was performed sequentially [107]. Meanwhile, wanted cells were identified based on three parameters: the number of detected genes ranged from 200 to 8000, the total number of UMIs was less than 5000, and the proportion of mitochondrial expression was lower than 25%. Cells not within this range were excluded. After retaining high-quality cell data, the common ‘Lognormalization’ method was used for expression normalization, and Harmony was used for data integration and batch effect correction [108].

### Cell clustering, visualization, and identification of cell types

Using the principal component analysis (PCA) method to perform cell clustering on normalized expression data [109], we then calculated the Euclidean distance between cells and embedded the cells into a shared-nearest-neighbor (SNN) graph. Finally, we used the Louvain method to cluster the ES cells and visualized the different cellular clusters using uniform manifold approximation and projection (UMAP plot) [110, 111].

DEG analysis of different cellular clusters was performed using the Wilcoxon rank-sum test in Seurat [112]. The upregulated DEGs in each cellular cluster were selected based on the criteria of log2FC ≥0.36, *P* value ≤0.01, and expression in more than 25% of the cells in the target cluster. Further analysis was conducted to identify cell types by examining the upregulated DEGs in cell clusters. These upregulated DEGs were compared with cell type-specific gene databases of other plant species (e.g., *A. thaliana*) (https://www.tobaccodb.org/pcmdb/homePage) to select specifically expressed marker genes (Table S7). Based on the functional annotation and the UMAP visualization of the expression of the homologous *G. biloba* marker genes, the cell types of various clusters in *G. biloba* ES were inferred.

### *In situ* RNA hybridization

After collecting the *G. biloba* seeds, immediately put them into *in situ* hybridization fixative solution (Wuhan Servicebio Technology Co., Ltd), and fix the tissues. Embed the tissues and cut them into 4-μm-thick sections, and bake the sections at 62°C for 2 h. Sequentially put the slices into two changes of BioDewax and Clear solution, 15 min each, then dehydrate in two changes of pure ethanol for 5 min each, then put into 85% alcohol and 75% alcohol, 5 min each, and soak them in diethyl pyrocarbonate (DEPC) water. Place the sections into a citrate buffer (pH 6.0) repair box and place it in a water bath at 90°C for 48 min. Digest with protease K (20 μg/ml) at 37°C for 10 min. Rinse with pure water and wash with PBS three times, 5 min each time. Add 3% methanol–H_2_O_2_, incubate at room temperature in the dark for 15 min, then place the sections in PBS (pH 7.4) and wash on a decolorization shaker 3 times, 5 min each time, to fully block endogenous peroxidase. Add prehybridization solution and incubate at 37°C for 1 h. Discard the prehybridization solution, add the probe hybridization solution, and hybridize overnight at 40°C. Wash off the hybridization solution and add the corresponding branch probe hybridization. After washing again, design probe sequences according to the marker gene sequences (Table S18), add the corresponding signal probe hybridization solution, and block with serum. Incubate with HRP-labeled mouse antidigoxigenin IgG (HRP-mouse anti-DIG IgG): discard the blocking solution and add HRP-mouse anti-DIG IgG. Incubate at 37°C for 50 mi, then wash with PBS four times, 5 min each time. Use DAB for color development and counterstain the cell nucleus with hematoxylin dye solution. After staining, dehydrate, seal, and mount the sections for microscopic examination and image acquisition.

### Pseudotime analysis of cellular clusters

Pseudotime trajectory analysis was conducted using the DDRTree algorithm in Monocle software [113]. Based on transcription factor relationships, the cells were pseudo-time ordered using the ‘orderCells’ function. Additionally, we utilized the ‘plot_cell_trajectory’ and ‘plot_pseudotime_heatmap’ functions to visualize the cell trajectories. Branch-dependent genes were analyzed using the ‘BEAM’ function. The ‘plot_genes_branched_heatmap’ was employed to illustrate gene expression bifurcations in two branches and visualize significant branch-dependent genes, followed by the identification of DEGs in different branches using the ‘differentialGeneTest’ function [114].

### Quasi-targeted metabolomic analysis

Using a scalpel, the SC tissues were precisely dissected from both the CK group and the JA treatment (200 mg/l JA) group. The SCs are identifiable as numerous clumps of white tissue embedded within the green fleshy episperm (ES). For each group, 100 mg of tissue was collected (with three biological replicates per group). The samples were ground to a fine powder in liquid nitrogen. Metabolites were then extracted by adding an extraction solution consisting of 80% methanol and 0.1% formic acid. The resulting supernatants, obtained after centrifugation, were subjected to LC–MS analysis [115]. LC–MS/MS analyses were performed using an ExionLC™ AD system (SCIEX) coupled with a QTRAP® 6500+ mass spectrometer (SCIEX) in Genedenovo (Guangzhou, China). Detection is performed using an Xselect HSS T3 column, 2.5 μm, 2.1 × 150 mm. The mobile phase A is 0.1% formic acid–water, and B is 0.1% formic acid–acetonitrile [116]. The column temperature is 50°C, and the flow rate is 0.4 ml/min. The chromatographic gradient is as follows: 0 min: 98% A and 2% B, 2 min: 98% A and 2% B, 15 min: 0% A and 100% B, 17 min: 0% A and 100% B, 17.1 min: 98% A and 2% B, 20 min: 98% A and 2% B. Mass spectrometry conditions in positive ion mode are as follows: curtain gas: 35 psi; collision gas: medium; ion spray voltage: 5500 V; temperature: 550°C; ion source gas 1: 60; ion source gas 2: 60. Mass spectrometry conditions in negative ion mode are as follows: curtain gas: 35 psi; collision gas: medium; ion spray voltage: −4500 V; temperature: 550°C; ion source gas 1: 60; ion source gas 2: 60. The analysis was conducted using a self-built database by Gene Denovo Biotechnology Co., Ltd (Guangzhou, China), employing the multiple reaction monitoring (MRM) mode to detect experimental samples. Quantification of compounds was based on Q3 (product ions), while identification was based on Q1 (precursor ions), Q3 (product ions), RT (retention time), DP (declustering potential), and CE (collision energy). The SCIEX OSV1.4 software was used to open the mass spectrometry files, integrate and correct chromatographic peaks, and filter chromatographic peaks based on settings such as a minimum peak height of 500, a signal-to-noise ratio of 5, and smoothing points of 1. The area of each chromatographic peak represents the relative content of the corresponding substance. Finally, all chromatographic peak area integration data were exported to obtain qualitative and quantitative results of the metabolites.

### GO and KEGG enrichment analysis

GO enrichment analysis of upregulated DEGs in seven cellular clusters was performed using the GO database (https://www.geneontology.org). KEGG enrichment analysis of DEGs and DEMs in two SC tissues (CK group and JA group) was performed using the KRGG database (https://www.kegg.jp). The p-values calculated were corrected by Bonferroni correction. GO and KEGG terms with corrected-pvalue ≤ 0.05 were defined as significantly enriched those terms in DEGs. GO and KEGG functional enrichment analysis can determine the main biological functions performed by DEGs

### Visualization of JA treated gene regulatory networks in secretory cavity

The *G. biloba* transcription factor-encoding genes were predicted by PlantTFDB (https://doi:10.18170/DVN/GHICUF). We identified enzyme-encoding genes (upregulated) and transcription factor genes that exhibit differential expression in SC cells under JA treatment (Tables S13 and S14). Correlation analysis was performed based on the expression levels of 19 enzyme-encoding genes and 51 transcription factor-encoding genes, and a molecular regulatory network was constructed and visualized using OmicShare tools (www.omicshare.com/tools/Home/Soft/cytoscape).

### RNA extraction and RT-qPCR analysis

Total RNA from the meristem cells, subepidermal cells, lignified cells/tracheid cells, parenchyma cells, SC cells, and epidermis cells of *G. biloba* episperms were separately isolated. In each sample, first-strand cDNA synthesis was performed following the manufacturer’s instructions (PrimeScriptTM RT Reagent Kit, TaKaRa, China). Real-time PCR was performed using a Perkin-Elmer 7000 thermal cycler with SuperReal PreMix Plus SYBR Green (TaKaRa, China) according to the manufacturer’s protocol. Reactions were performed using 10 μl of 2× SuperReal PreMix Plus (with SYBR Green I), 0.8 μl of 10 μM each primer, 4 μl of diluted cDNA, and nuclease-free water to a final volume of 20 μl. PCR reaction program was as follows: 95°C for 15 min, then 95°C for 10 s, 60°C for 30 s, 40 cycles [117]. The raw data obtained from the PCR experiment was subjected to analysis using the Light Cycler software. Subsequently, the expression level was normalized according to the *GAPDH* gene of *Ginkgo* (accession NO. D27166). The 2^−ΔΔCt^ method was utilized to calculate the relative expression level [118]. The mean of three independent biological replicates with internal repeats was used to represent the RT-qPCR data.

### Bioinformatics analysis

The CDS sequence and the amino acid sequence encoded by *GbWRKY35* were obtained from the *Ginkgo* genome website database (https://ginkgo.zju.edu.cn/genome/). The secondary structure was predicted online using the Uniprot website (https://www.uniprot.org). The conserved domains of the transcription factor were predicted online using the SMART website (https://smart.embl.de/). The hydrophobicity of GbWRKY35 was predicted online using the Protscale website (https://web.expasy.org/cgi-bin/protscale/protscale.pl?1). The transmembrane regions were analyzed and the signal peptides were predicted online using the TMHMM website (http://www.cbs.dtu.dk/services/TMHMM/) and the SignalP website (https://services.healthtech.dtu.dk/services/SignalP-5.0/), respectively. An online blast comparison was conducted with the *Arabidopsis* database website (https://www.arabidopsis.org/), and the homologous amino acids were analyzed by multiple sequence alignment using DNAMAN3.0 software.

### cDNA library construction for yeast two-hybridization screening

Fifteen milliliters of CTAB extraction buffer was preheated to 65°C in a water bath. Subsequently, 2–3 g of frozen *G. biloba* leaf, seed, and episperm tissues were ground into a fine powder using liquid nitrogen. The powdered tissue was transferred to a centrifuge tube containing the preheated CTAB extraction buffer, vortexed vigorously for 30 seconds, and incubated in a 65°C water bath for 5–10 min. An equal volume of chloroform/isopropanol was added to the mixture, followed by thorough vortexing. The sample was then centrifuged at 10000 rpm for 15 min at room temperature.

The supernatant was carefully transferred to a new RNase-free centrifuge tube. RNA was precipitated adding 8 M LiCl to a final concentration of 2 M (equivalent to a 1/3 volume of the supernatant), and incubated overnight at 4°C (for up to 16 h). Following incubation, the sample was centrifuged at 12000 rpm at 4°C for 30 min. The supernatant was discarded, and the RNA pellet was washed with 10 ml of 70% ethanol (RNase-free). The sample was centrifuged again at 12000 rpm at 4°C for 5 min and the supernatant was discarded. Finally, the RNA pellet was air-dried in a laminar flow hood and dissolve it in 500 μl of DEPC-treated water to obtain total RNA.

According to the CloneMiner manual, first-strand and second-strand cDNA were synthesized from the extracted total RNA. The double-stranded cDNA was then ligated to the three-frame *attB1* recombination adapter [attB1 Adapter (1 μg/μl) F 4.5 μl, attB1 Adapter (1ug/μl) R 4.5 μl, 10 × NEB Buffer 2 1 μl] by incubating at 95°C for 5 min. The sample was then centrifuged at 10000 rpm for 15 min at room temperature.

The adapted cDNA was then incubated with T4 DNA Ligase (1 U/μl) in 1× ligation buffer to facilitate circularization. The circularized cDNA was size-fractionated to remove short fragments, and the desired fractions were collected. A BP recombination reaction was performed by mixing the size-selected cDNA with the pDONR222 vector (200 ng/μl) and BP Clonase® II enzyme mix. The resulting reaction product was transformed into *Escherichia coli* DH10B competent cells. The primary cDNA library was assessed for capacity, recombination rate, and insert fragment length.

A qualified uncut library plasmid (300 ng/μl) was used for an LR recombination reaction with the pGADT7-DEST vector (300 ng/μl) and LR Clonase® II enzyme mix in ddH₂O. The reaction product was transformed into *E. coli* DH10B to generate a secondary library. The secondary library was similarly validated for capacity, recombination rate, and insert size. Finally, the validated secondary library plasmid was transformed into the Y187 yeast strain to create a working stock for yeast two-hybrid screening.

### Yeast one-hybrid assay

The coding sequence of the *Gb_25334* was synthesized using a whole-gene synthesis method and subsequently cloned into the pGADT7 vector, resulting in a fusion protein with the GAL4 activation domain (AD). Similarly, the putative promoter sequences for *Gb_18621* and *Gb_31209*, defined as the 1500-bp region upstream of the respective transcription start sites, were synthesized and independently cloned into the pHIS2 reporter vector, upstream of the *HIS* reporter gene. The resultant bait and prey constructs were co-transformed in pairs into the *Saccharomyces cerevisiae* reporter strain Y187. This generated two distinct yeast strains for analysis: one containing pHIS2-Gb_18621*pro* + pGADT7-*Gb_25334*, and the other containing pHIS2-Gb_31209*pro* + pGADT7-*Gb_25334*. Prior to the interaction assay, a background screening was essential to establish the appropriate selective conditions. The auto-activation potential of each promoter–reporter construct was tested by co-transforming them with the empty pGADT7 vector. The strains were plated on synthetic dropout (SD) medium lacking histidine and supplemented with 3-amino-1,2,4-triazole (3AT), a competitive inhibitor of the HIS3 gene product. The minimal inhibitory concentrations of 3AT required to fully suppress background growth were determined to be 100 mM for the pHIS2-Gb_18621*pro* strain and 40 mM for the pHIS2-Gb_31209*pro* strain, confirming no intrinsic activation of the *HIS3* reporter. The experimental yeast strains, containing both the pGADT7-*Gb_25334* prey and the respective pHIS2-promoter bait constructs, were subsequently assayed for protein–promoter interaction. The strains were cultured on SD/−His plates supplemented with the predetermined, stringent concentrations of 3AT (100 mM for Gb_18621*pro* and 40 mM for Gb_31209*pro*). A physical interaction between the Gb_25334-AD fusion protein and the target promoter was inferred based on the growth status of the yeast, which would indicate successful activation of the *HIS3* reporter gene under these selective conditions.

### Dual-luciferase reporter assay

The plant material used in this experiment, *Nicotiana benthamiana*, was grown under the following conditions: in a greenhouse at 22°C–25°C (in the light) and 18°C–20°C (in the dark), with a light period of 14 h. The plants were used for *Agrobacterium*-mediated transient transformation experiments after growing for 4–6 weeks (at the five-leaf stage). The previously synthesized and cloned *Gb_25334* (*GbWRKY35*) gene and *Gb_31209* (*GbAAE16*) promoter were respectively recombined into the vectors pBinGFP2 and pGreenII 0800-LUC. The recombinant plasmids were transformed into *E. coli* TOP10, and the sequences of the recombinant plasmids were sequenced to verify the correctness of the target sequences. The correctly recombined plasmid vectors were then transformed into *Agrobacterium* GV3101, and the successfully transformed *Agrobacterium* was injected into tobacco leaves and marked. The interaction between the samples to be detected was analyzed using the ChemiDoc imaging system. The leaves were placed in the ChemiDoc imager to detect the luminescence, and the appropriate exposure intensity was adjusted and the phenotype was recorded by taking pictures. The infected leaves were crushed to extract the supernatant protein, and the reaction solution with the corresponding concentration of luciferase substrate was added. The reaction was incubated, and the fluorescence intensity was quantitatively analyzed using a microplate reader to directly analyze the interaction intensity.

### Two-hybrid library screening using yeast mating

The gene *Gb_25334* was constructed into the pGBKT7 vector as a bait, and the pGBKT7-*Gb_25334* and pGADT7 were co-transformed into AH109 yeast. By detecting the growth of the yeast on SD-TL, SD-TLH, SD-TLHA, and SD-TLHA + X-α-gal plates, it was found that there was an auto-activation phenomenon. Defective plates of SD-TLH + 0 mM 3AT, SD-TLH + 2.5 mM 3AT, SD-TLH + 5 mM 3AT, SD-TLH + 10 mM 3AT, SD-TLH + 20 mM 3AT, SD-TLH + 30 mM 3AT, SD-TLH + 40 mM 3AT, SD-TLH + 50 mM 3AT, SD-TLH + 75 mM 3AT, and SD-TLH + 100 mM 3AT were prepared. The pGBKT7-Gb_25334 and pGADT7 were co-transformed into AH109 yeast and cultured on the above plates. It was determined that the 3AT inhibitory concentration on the plate was 2.5 mM to inhibit the auto-activation phenomenon. The verified correct pGBKT7-*Gb_25334* clone and the Ginkgo cDNA library glycerol bacteria were co-cultured and co-coated on the SD-TLH + 2.5 mM 3AT plate to observe the colony growth. All the PCR amplifications of the successfully grown positive clone colonies were carried out. After Seqman and BLAST comparisons, the target gene sequence of Ginkgo with interaction function was finally obtained. The positive clone colonies of the successful sequencing were streaked on the SD-TLH + 2.5 mM 3AT defective plate, and the transformants grown were suspended in sterile water to adjust the OD600 = 0.2 and spotted on SD-TL, SD-TLH + 2.5 mM 3AT, SD-TLHA +2.5 mM 3AT, and SD-TLHA +2.5 mM 3AT + X-α-gal defective plates. They were incubated at 30°C for 3–4 days for reverse verification.

## Supplementary Material

Web_Material_uhag064

## Data Availability

Raw reads have been deposited as a BioProject under accession PRJNA1214489.
