# Supplementary material for: Single-nucleus sequencing and spatial metabolomics analysis reveal the regulatory mechanism of ginkgolic acid biosynthesis in the episperm of Ginkgo biloba
Source: Hortic Res. 2026 Feb 28;13(6):uhag064. doi: 10.1093/hr/uhag064 (PMC13249510; doi:10.1093/hr/uhag064)
Supplement: Web_Material_uhag064 [file web_material_uhag064.zip › Supplement figure(Revised).docx]

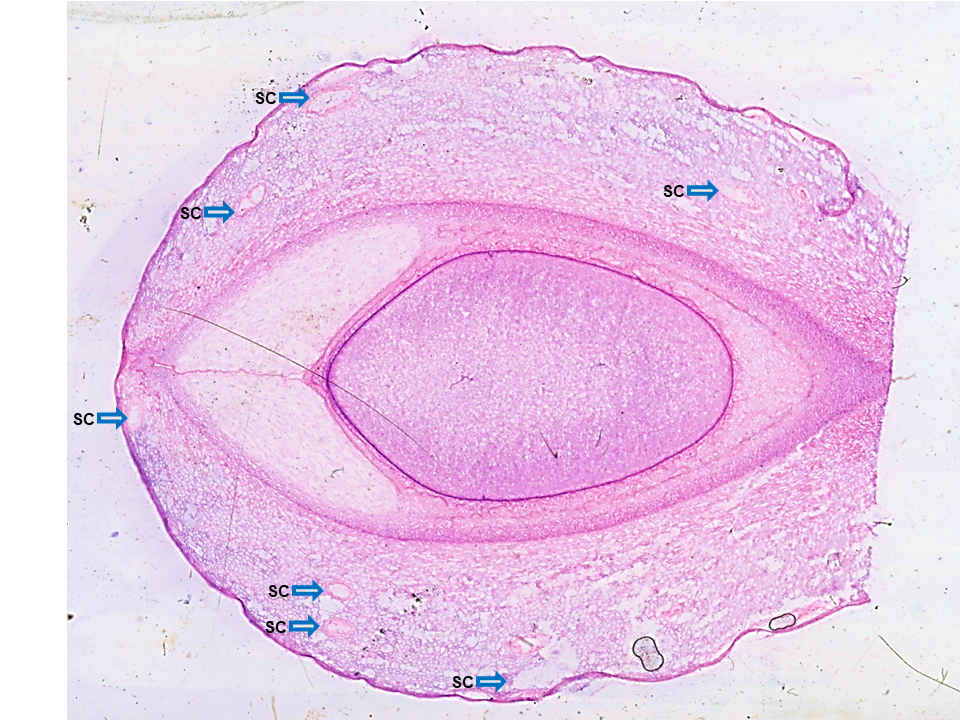


**Supplementary Figure 1. Cellular microscopic observation in the longitudinal sections of *G. biloba* seed through frozen sectioning and staining with HE (hematoxylin-eosin).** SC represents secretory cavities.


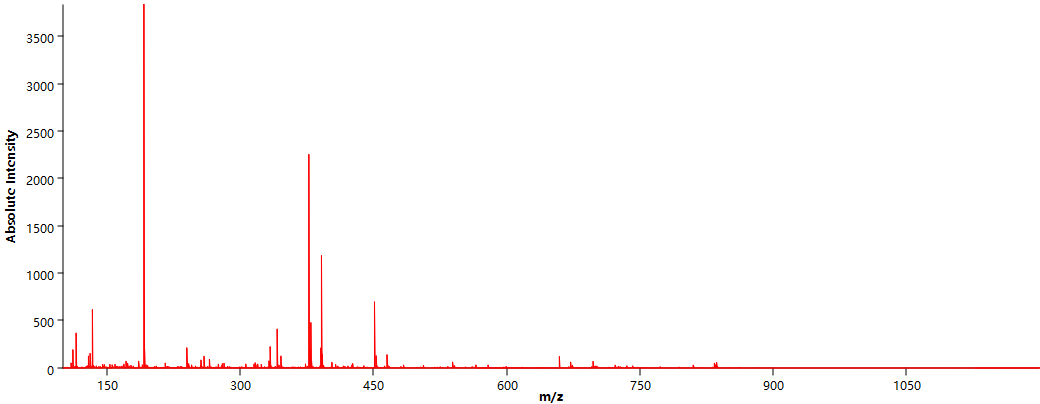


**Supplementary Figure 2. Representative single-pixel matrix-assisted laser desorption/ionization (MALDI) ToF mass spectra acquired from a cross section of the *G. biloba* seed.**

**
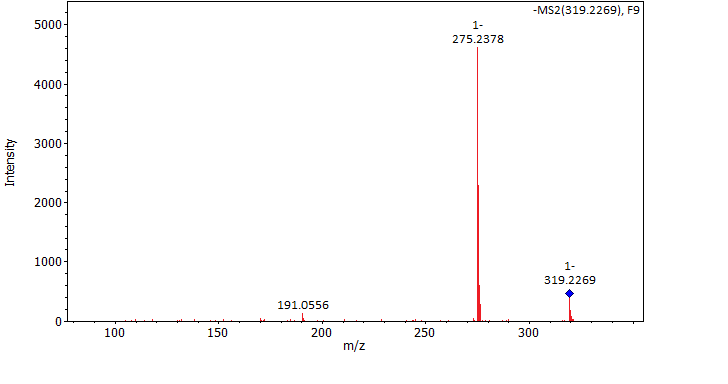

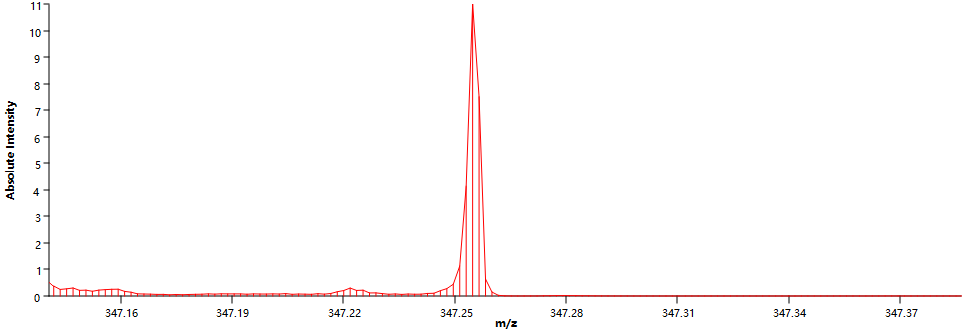
**

**GA 13:0 GA 15:0**

**
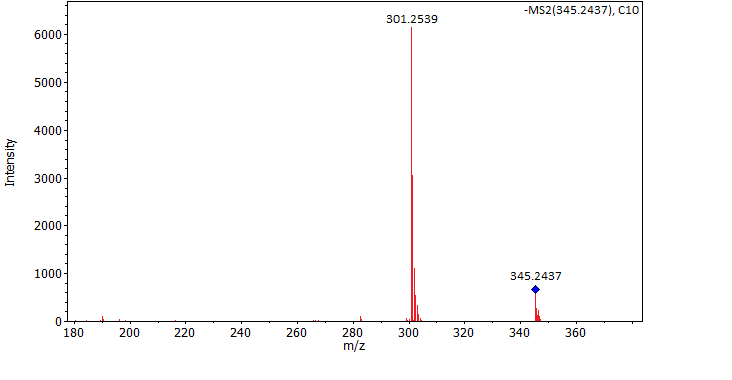

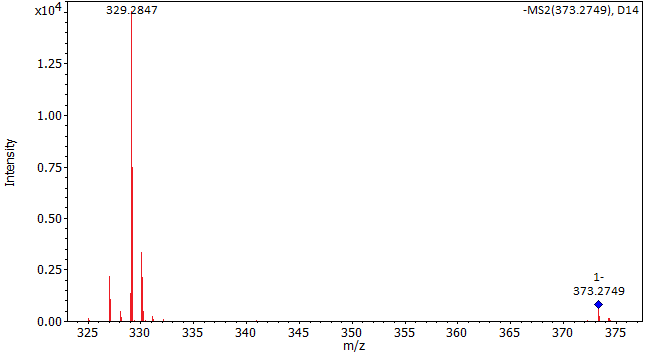
**

**GA 15:1 GA 17:1**

**
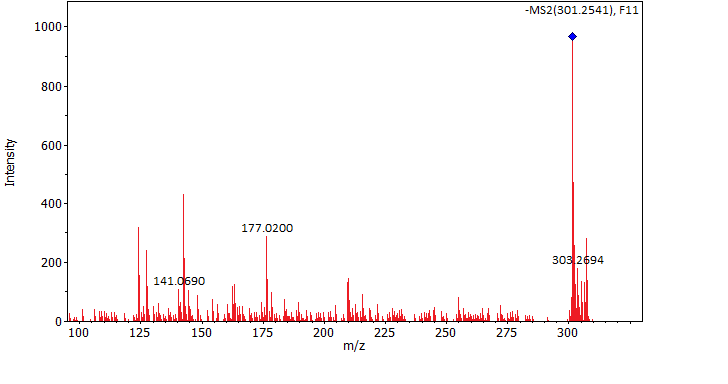

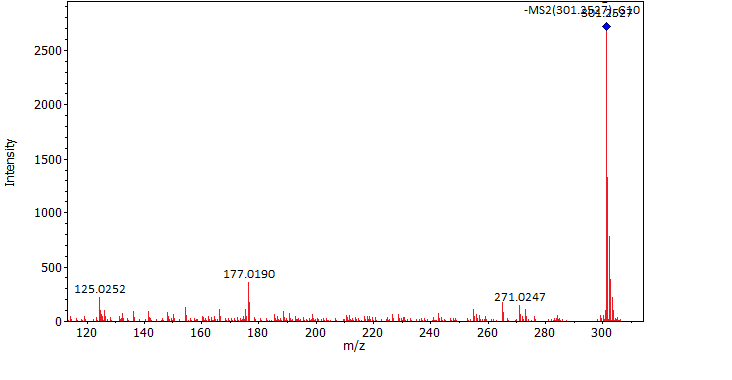
**

**PDP CA 15:1**

**Supplementary Figure 3. I Identification of ginkgolic acids in the *G. biloba* seed accomplished by MALDI LIFT ToF/ToF MS/MS.** GA 13:0 is ginkgolic acid C13:0, common name as 2-Hydroxy-6-tridecylbenzoic acid. GA 15:0 is ginkgolic acid C15:0 (also named anacardic acid), common name as 2-Hydroxy-6-pentadecylbenzoic acid. GA 15:1 is ginkgolic acid C15:1, common name as 2-hydroxy-6-[(8E)-pentadec-8-en-1-yl]benzoic acid. GA 17:1 is ginkgolic acid C17:1, common name as 2-(10-Heptadecenyl)-6-hydroxybenzoic acid. PDP is cardanol C15:0, common name as 3-Pentadecylphenol. CA 15:1 is cardanol C15:1, common name as Cardanolmonoene.


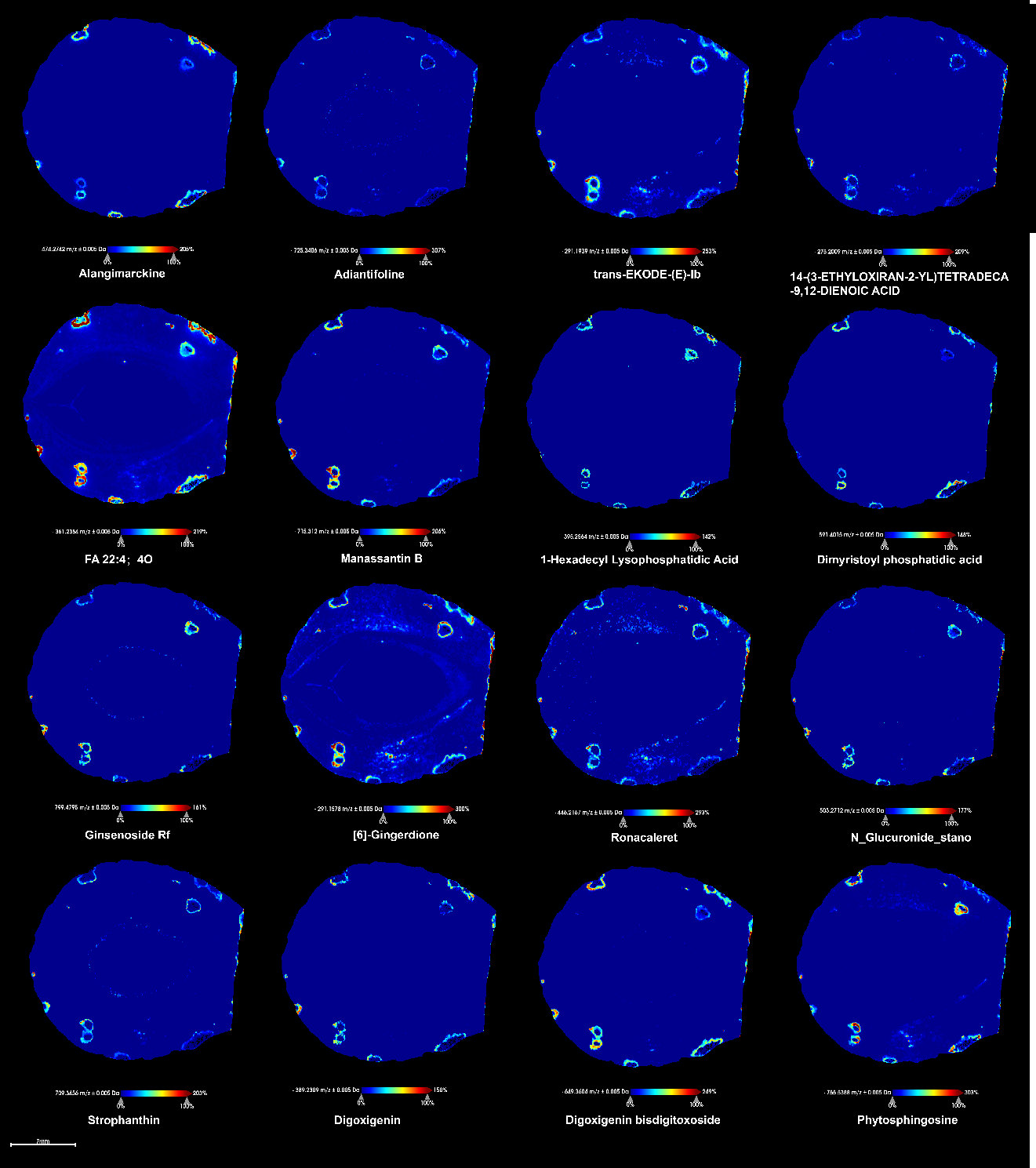


**Supplementary Figure 4. Different metabolites accumulate in the secretory cavities of the episperm** alkaloid compounds (Alangimarckine and Adiantifoline), fatty acyl compounds (trans-EKODE-(E)-Ib, 14-(3-ETHYLOXIRAN-2-YL)TETRADECA-9,12-DIENOIC ACID and FA 22:4), furanoid lignan compounds (Manassantin B), glycerophospholipid compounds (1-Hexadecyl Lysophosphatidic Acid, Dimyristoyl phosphatidic acid and Ginsenoside Rf), phenol ether compounds ([6]-Gingerdione), phenylpropanoic acid compounds (Ronacaleret), steroid compounds (N_Glucuronide_stano, Strophanthin and Digoxigenin) and sphingolipid compound (Phytosphingosine). Different colors represent different relative content, with darker shades of blue indicating lower content and darker shades of red indicating higher content.

**Standardized total ginkgolic acids**


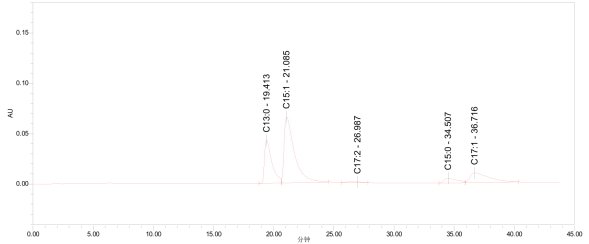

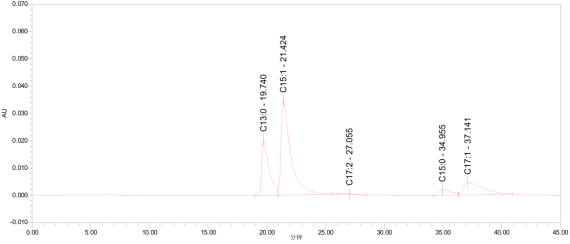


**2000 ppm 1000 ppm**


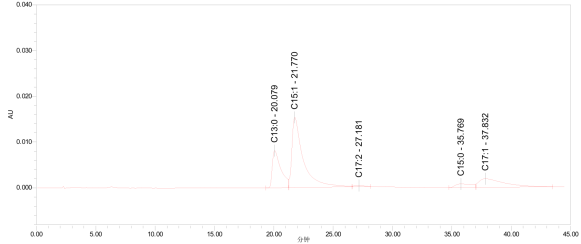

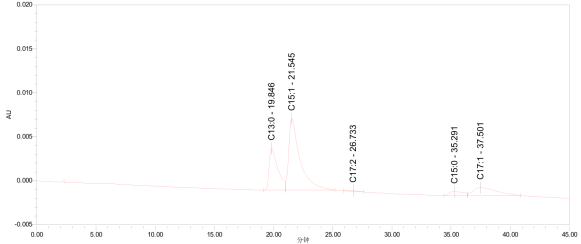


**500 ppm 250 ppm**


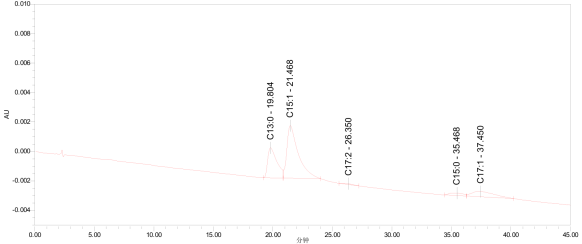


**125 ppm**

**Total ginkgolic acids in samples**

**CK JA 100**


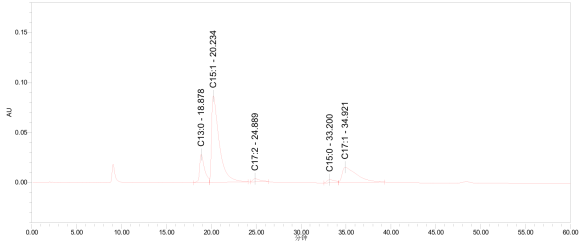

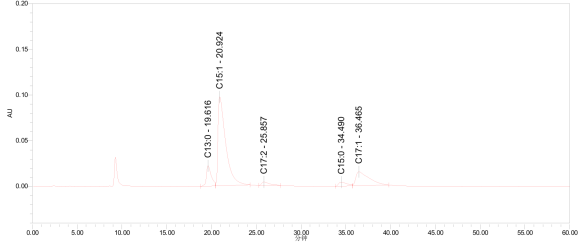


**JA 200 JA 300**


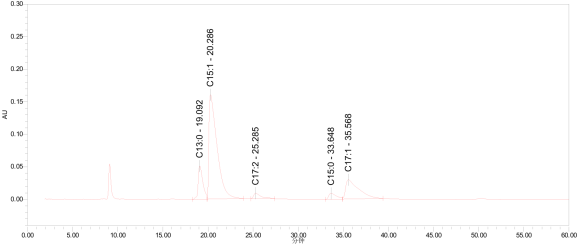

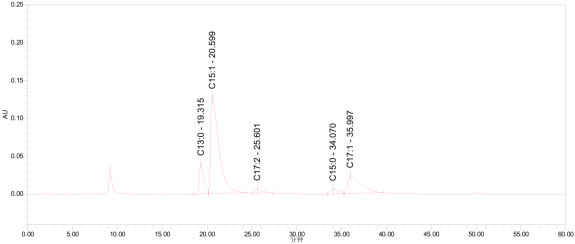


**JA 400**


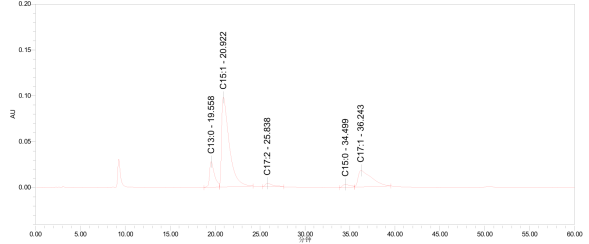


**Supplementary Figure 5. Chromatogram of total ginkgolic acids by HPLC.**

**
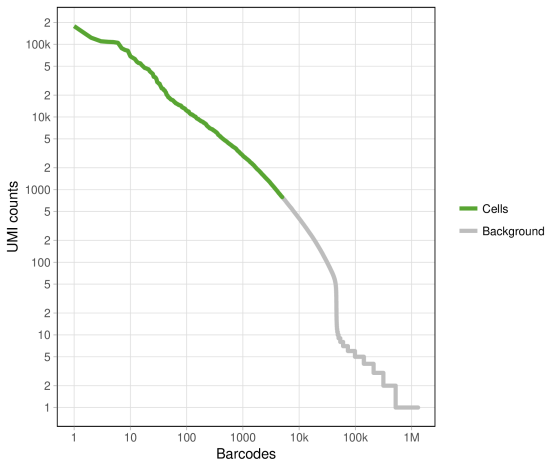

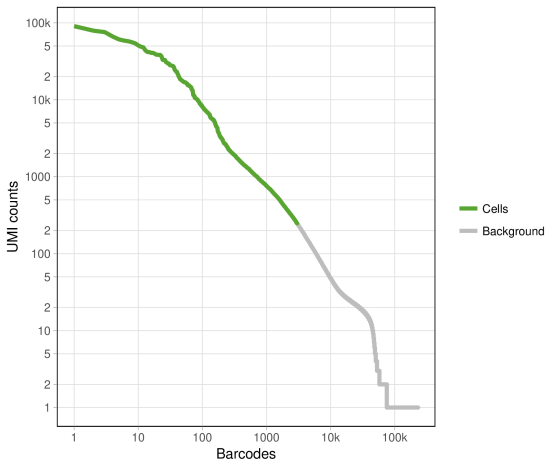
**

**CK JA**

**Supplementary Figure 6. Valid cell identification**

**
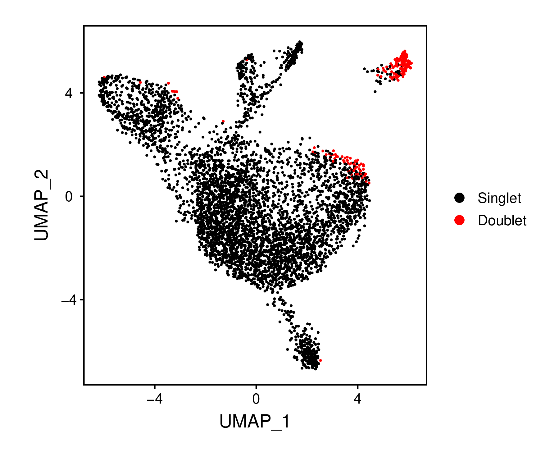

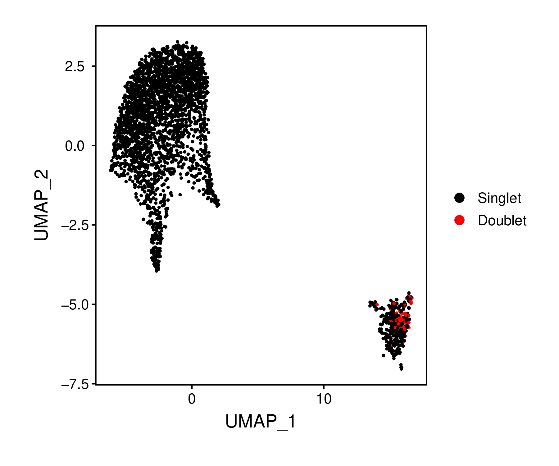
**

**CK JA**

**Supplementary Figure 7. Multi-cell UMAP plot.**

**
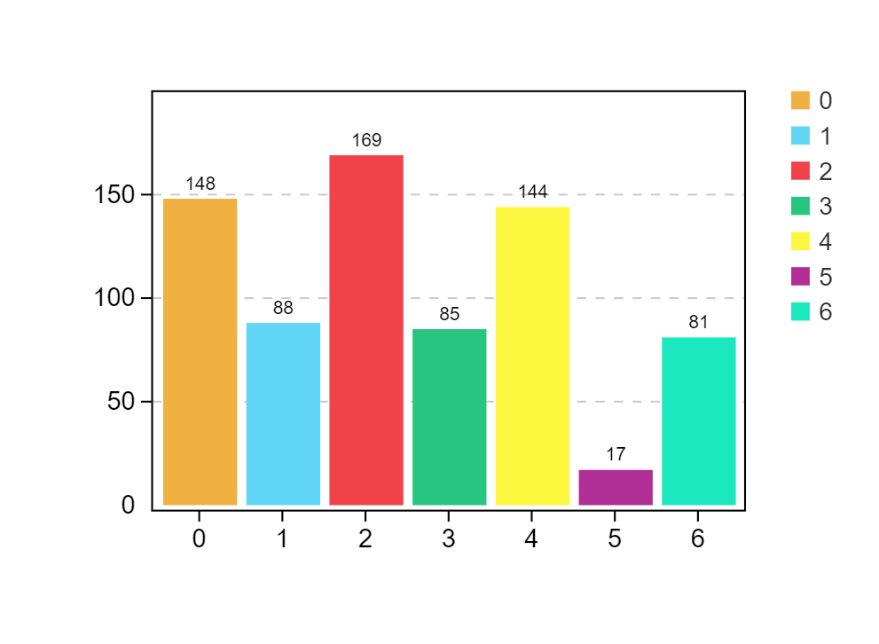
**

**Supplementary Figure 8. Statistics of gene numbers with upregulated expression in cellular cluster.**


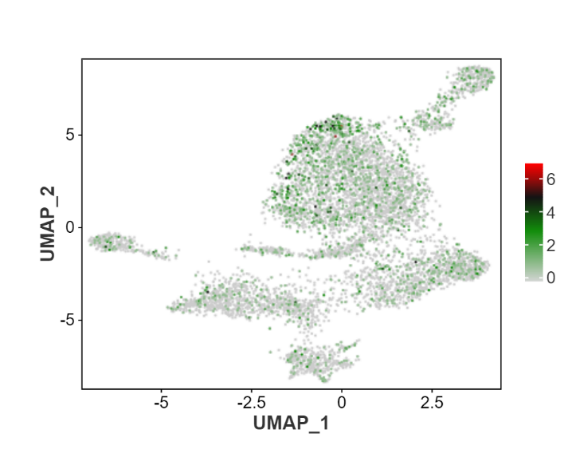

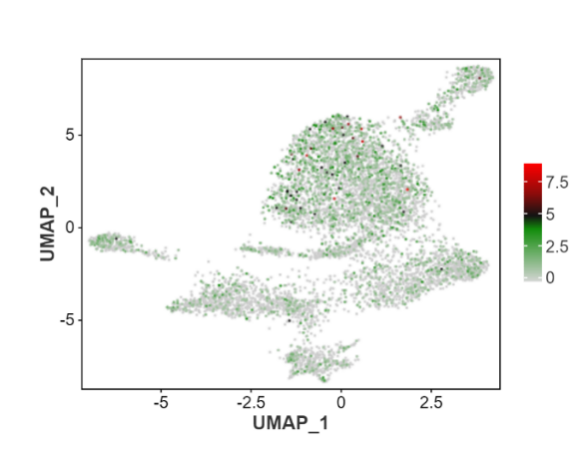


**Gb_01521 (ARF) Gb_05796 (HMGB3)**


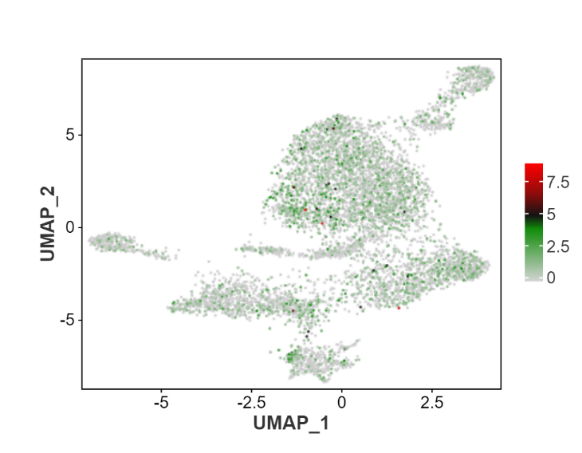

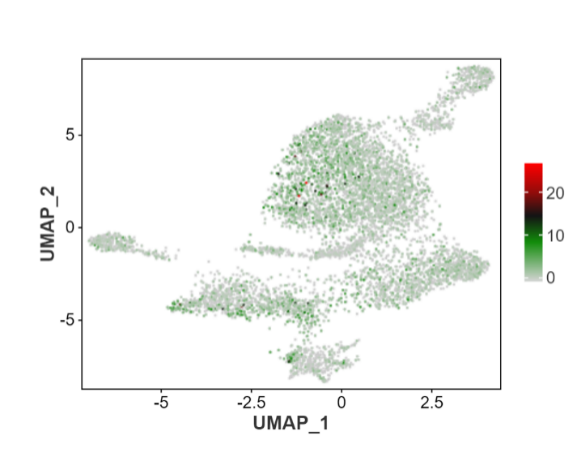


**Gb_05772 (ERF) Gb_17413 (PIP2)**


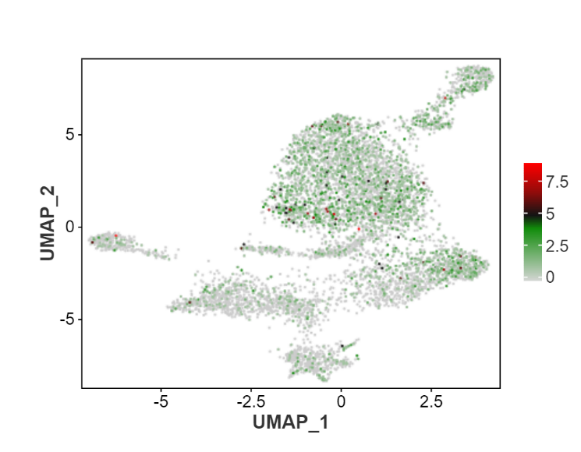


**Gb_24881 (KINB2)**

**Supplementary Figure 9. UMAP plot showing the representative marker genes of cellular cluster 0.**

**
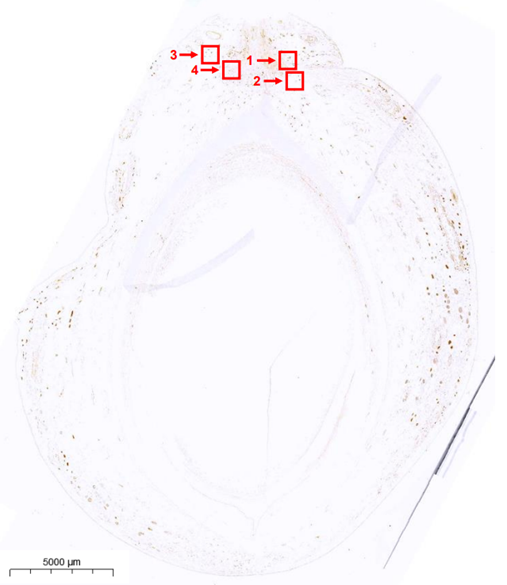
**

**
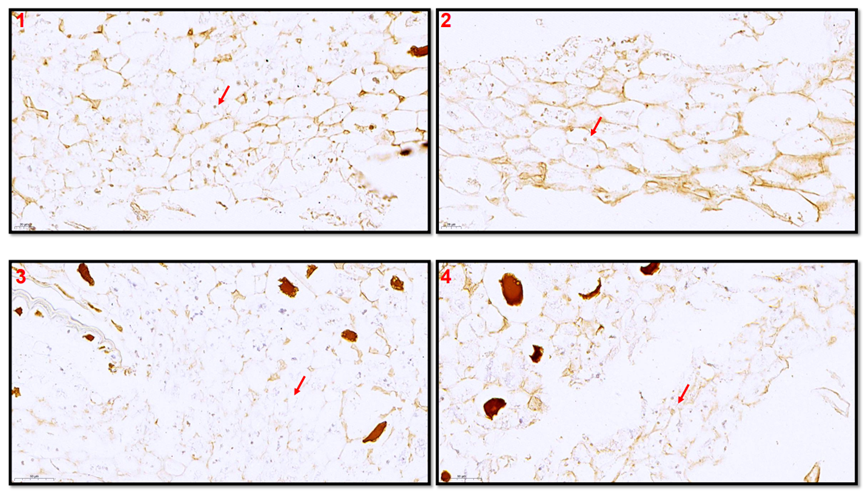
**

**Supplementary Figure 10. RNA *in situ* hybridization of *Gb_05796* (*HMGB3*), which is marker gene for the meristematic cells (MCs).**

* The Red arrows indicate cells with successful RNA in situ hybridization. Hematoxylin stains the cell nucleus blue, and DAB shows cell nucleus as brownish-yellow. The darker the brownish-yellow color of the cell nucleus, the higher the expression level.


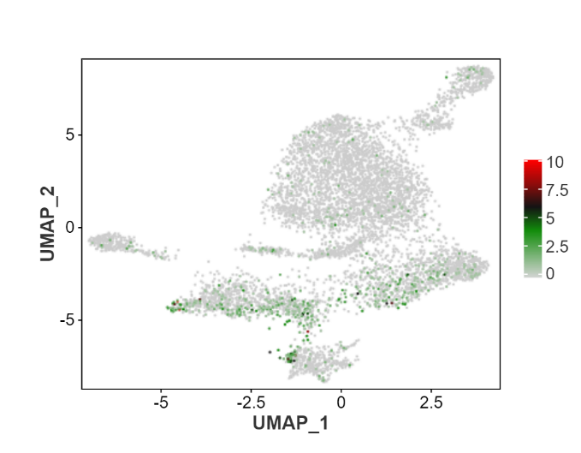

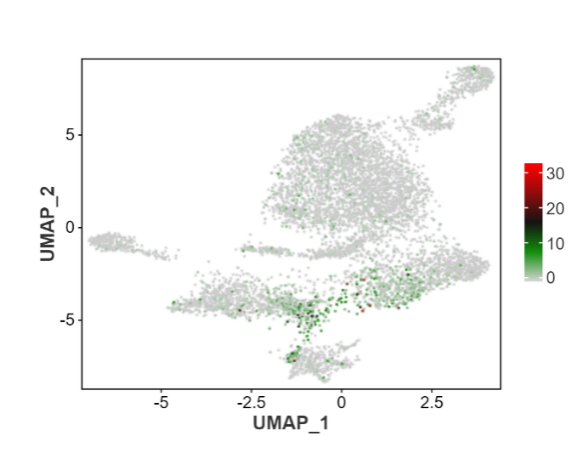


**Gb_06452 (ERG3) Gb_09283 (PER12)**


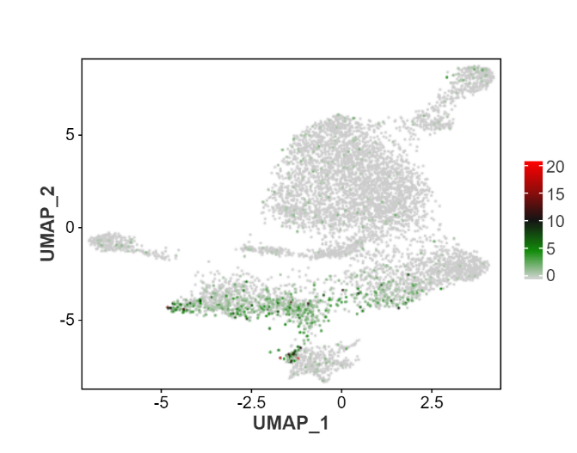

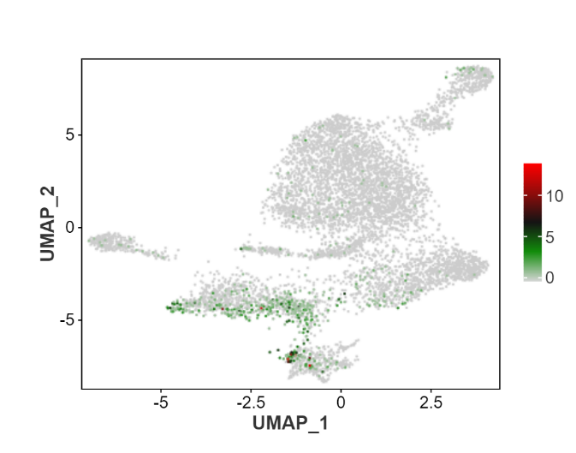


**Gb_15664 (SAUR10) Gb_20903 (IAMT1)**


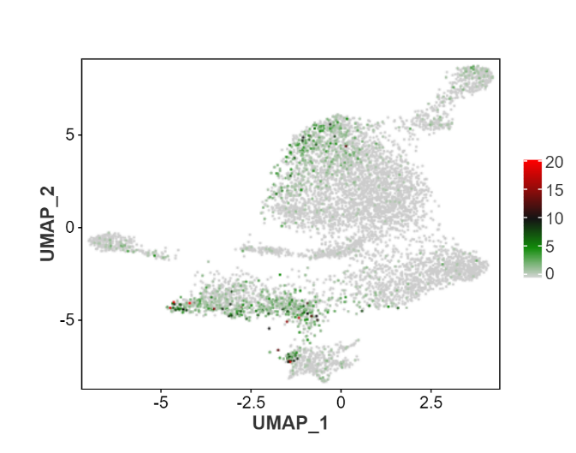


**Gb_26584 (TIP1)**

**Supplementary Figure 11. UMAP plot showing the representative marker genes of cellular cluster 1.**

**
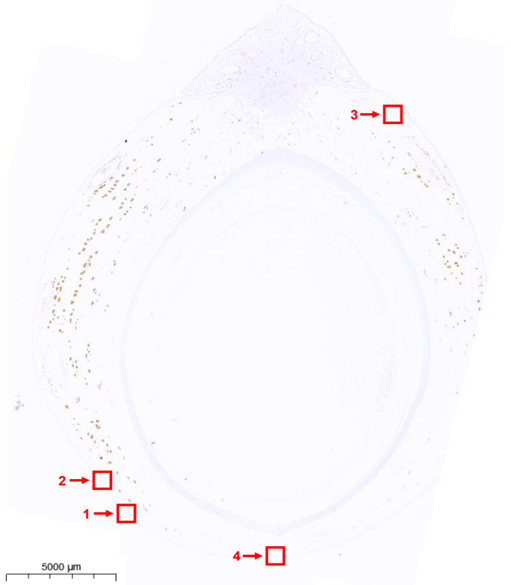
**

**
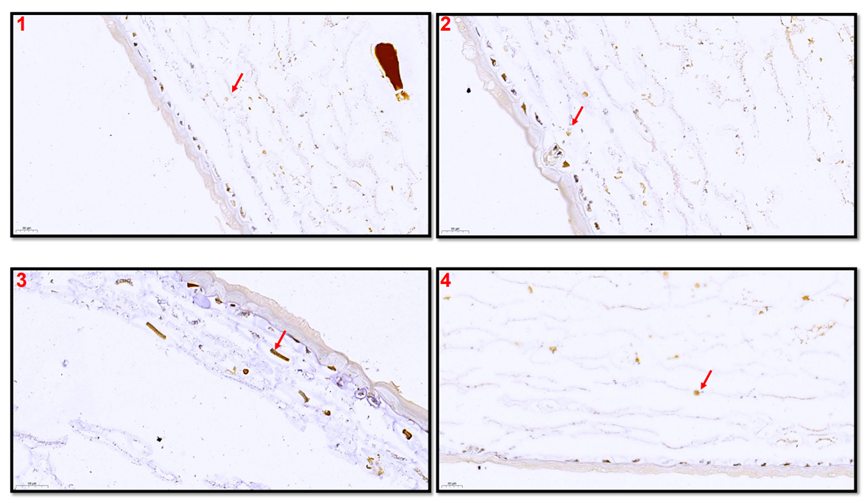
**

**Supplementary Figure 12. RNA *in situ* hybridization of *Gb_06452* (*ERG3*), which is marker gene for the subepidermal cells (SECs).**

*The red arrows indicate cells with successful RNA in situ hybridization. Hematoxylin stains the cell nucleus blue, and DAB shows cell nucleus as brownish-yellow. The darker the brownish-yellow color of the cell nucleus, the higher the expression level.


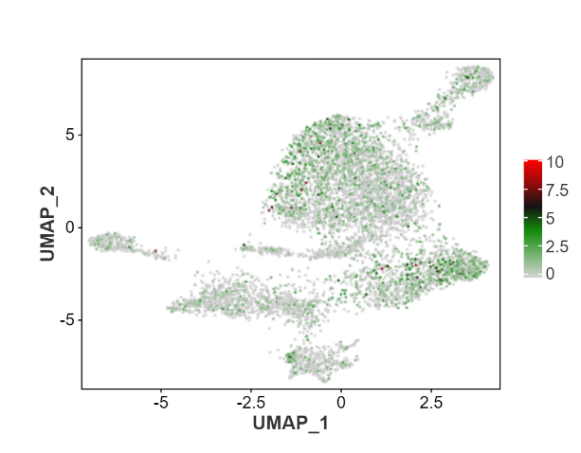

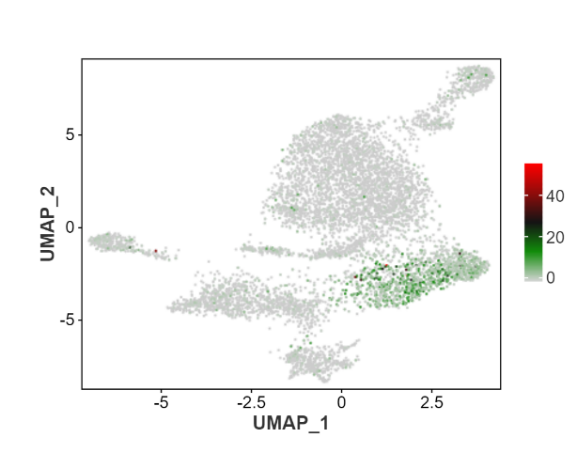


**Gb_01128 (RNS1) Gb_01519 (CHS)**


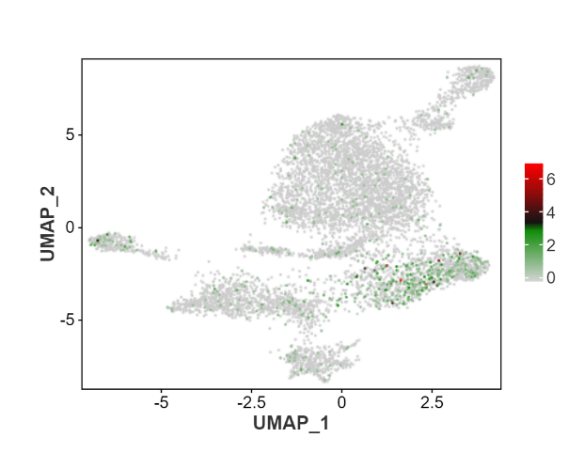

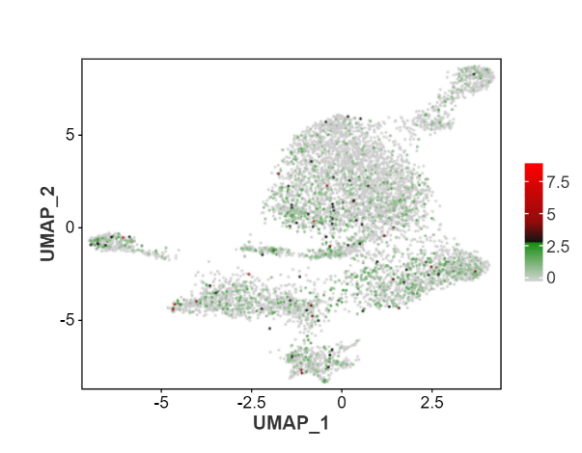


**Gb_07234 (CHI) Gb_17602 (SWEET1)**


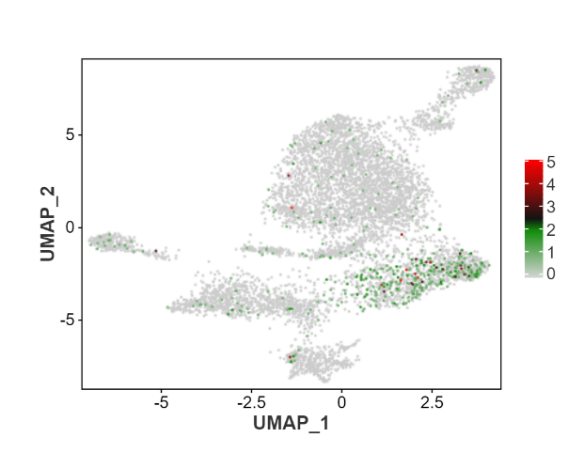


**Gb_34744 (XCP1)**

**Supplementary Figure 13. UMAP plot showing the representative marker genes of cellular cluster 2.**

**
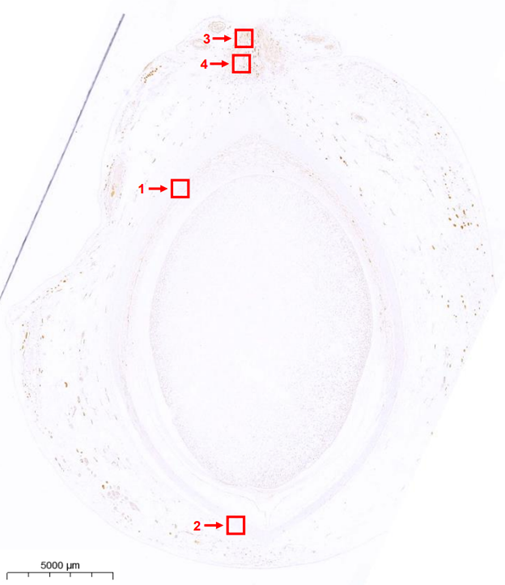
**

**
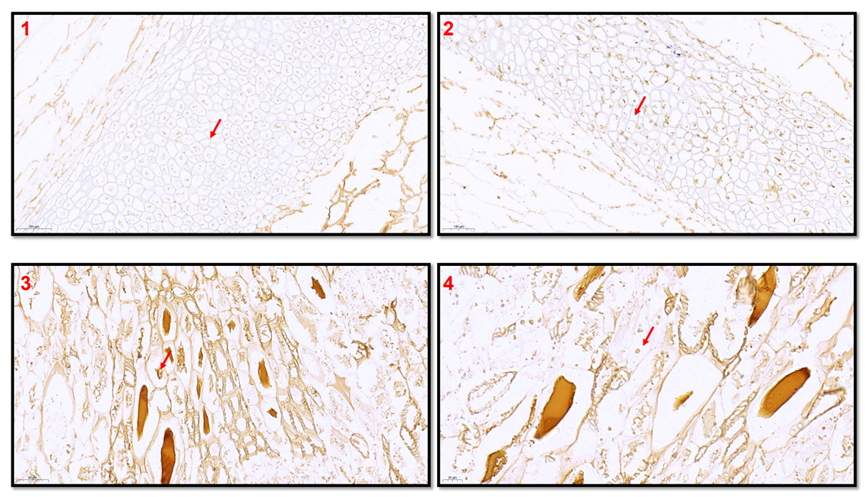
**

**Supplementary Figure 14. RNA *in situ* hybridization of *Gb_34744* (*XCP1*), which is marker gene for the lignified cells (LCs).**

* The red arrows indicate cells with successful RNA in situ hybridization. Hematoxylin stains the cell nucleus blue, and DAB shows cell nucleus as brownish-yellow. The darker the brownish-yellow color of the cell nucleus, the higher the expression level.


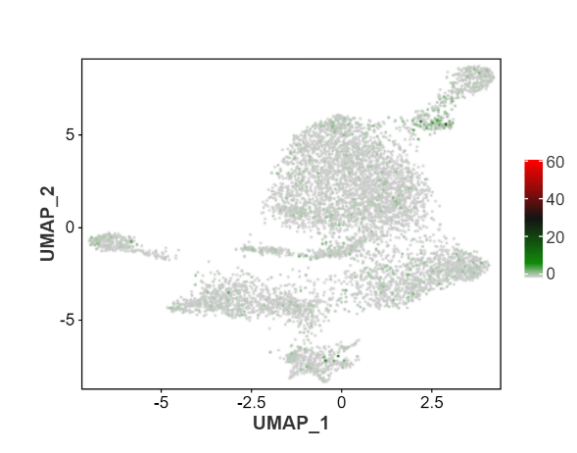

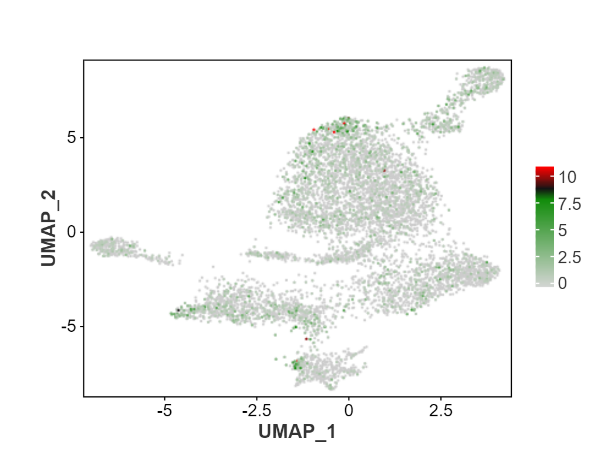


**Gb_07962 (TUBA) Gb_09532 (CYS6)**


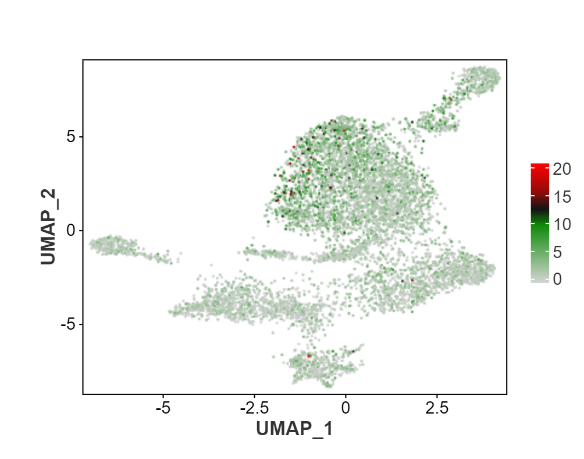

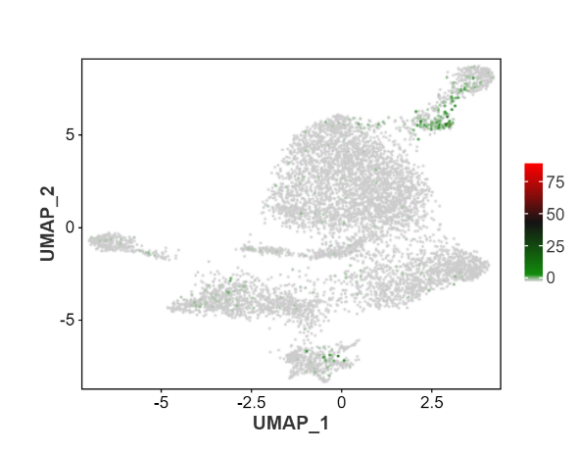


**Gb_16865 (UBC28) Gb_17655 (GLP7)**


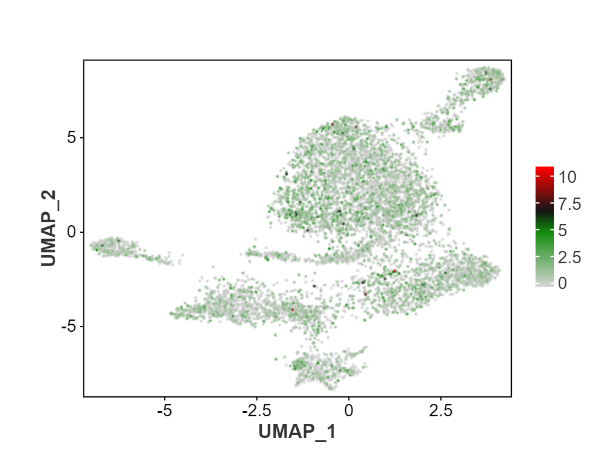


**Gb_21641 (UBC2)**

**Supplementary Figure 15. UMAP plot showing the representative marker genes of cellular cluster 3.**

**
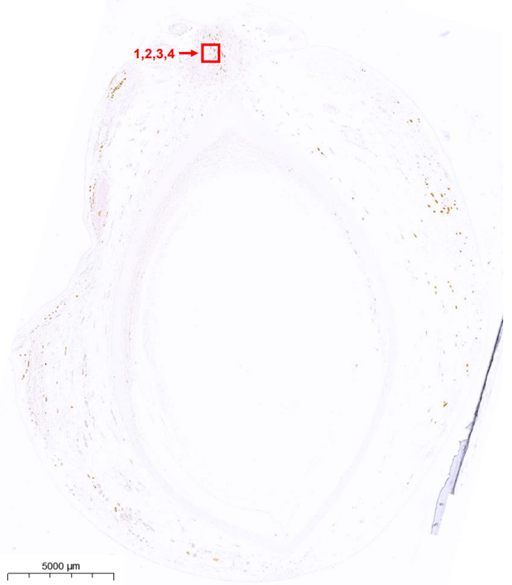
**

**
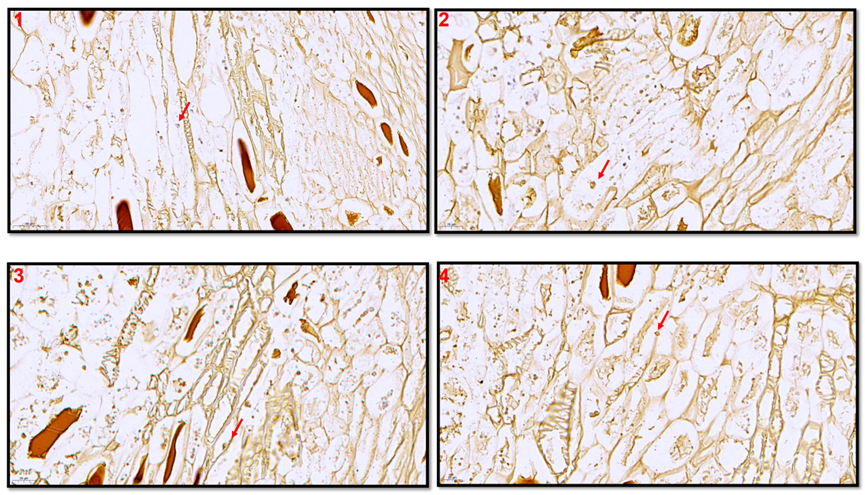
**

**Supplementary Figure 16. RNA *in situ* hybridization of *Gb_17655* (*GLP7*), which is marker gene for the** **tracheid cells (TCs).**

* The red arrows indicate cells with successful RNA in situ hybridization. Hematoxylin stains the cell nucleus blue, and DAB shows cell nucleus as brownish-yellow. The darker the brownish-yellow color of the cell nucleus, the higher the expression level.


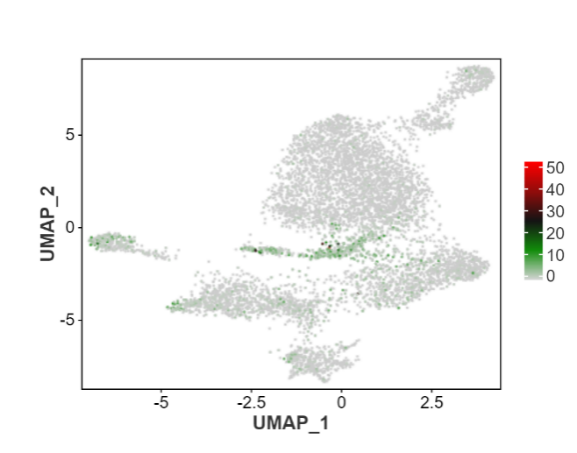

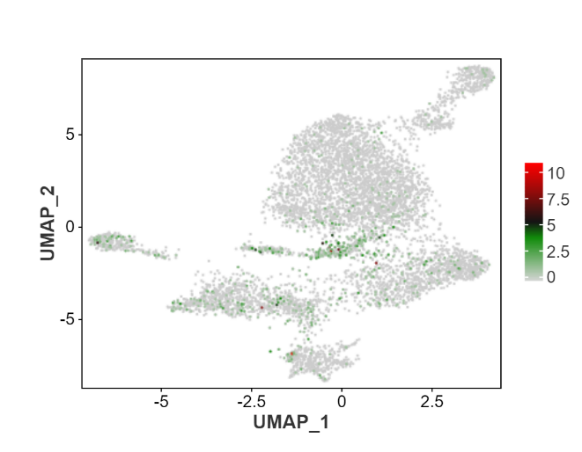


**Gb_00380 (RBCS) Gb_10163 (CAB8)**


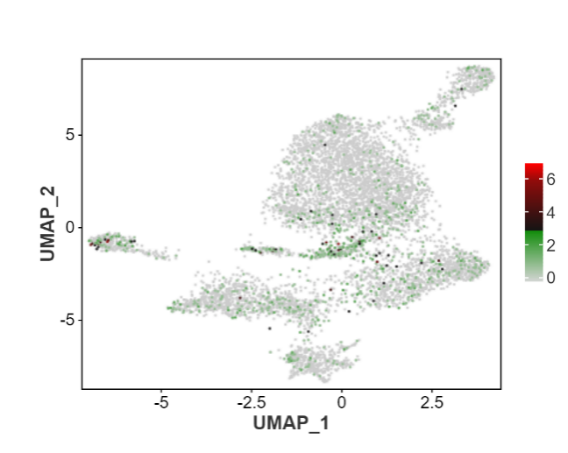

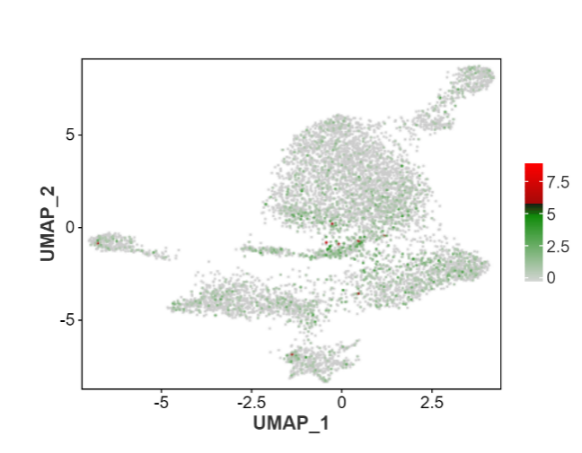


**Gb_18375 (CAB6) Gb_27633 (CAP10)**


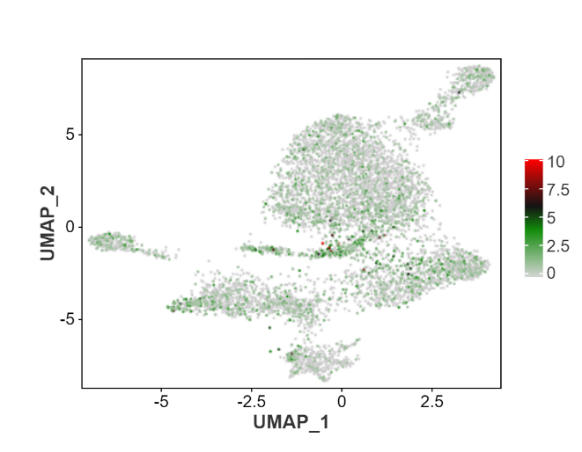


**Gb_38595 (LHCB4)**

**Supplementary Figure 17. UMAP plot showing the representative marker genes of cellular cluster 4.**

**
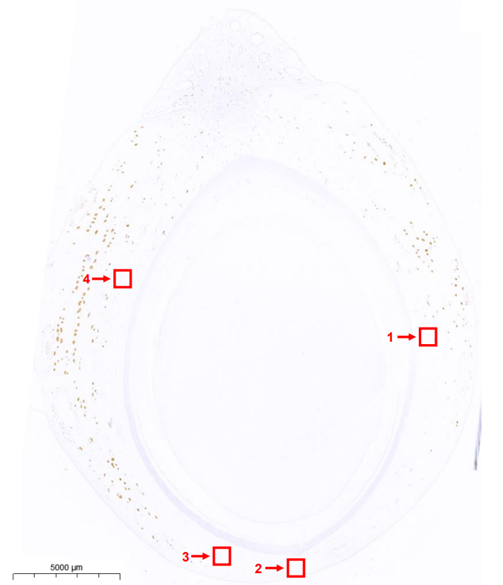
**

**
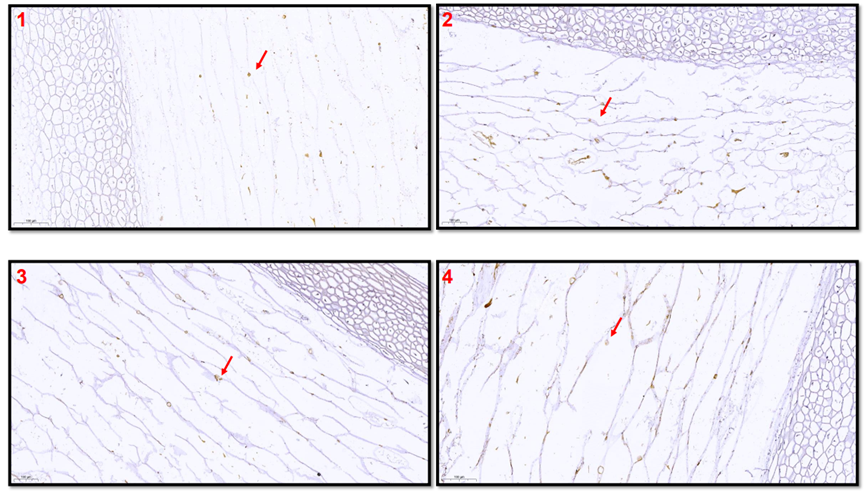
**

**Supplementary Figure 18. RNA *in situ* hybridization of *Gb_10163* (*CAB8*), which is marker gene for the** **parenchyma cells (PCs).**

* The red arrows indicate cells with successful RNA in situ hybridization. Hematoxylin stains the cell nucleus blue, and DAB shows cell nucleus as brownish-yellow. The darker the brownish-yellow color of the cell nucleus, the higher the expression level.


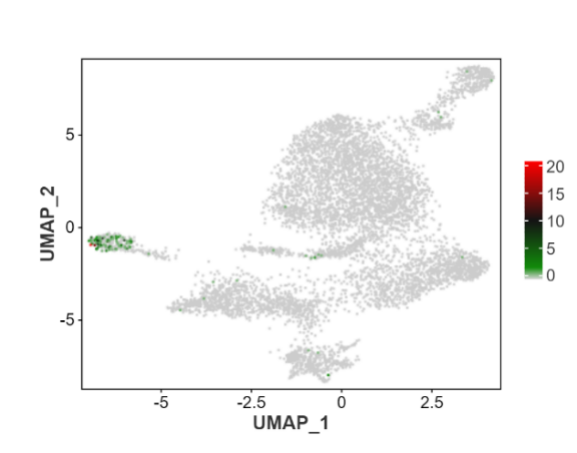

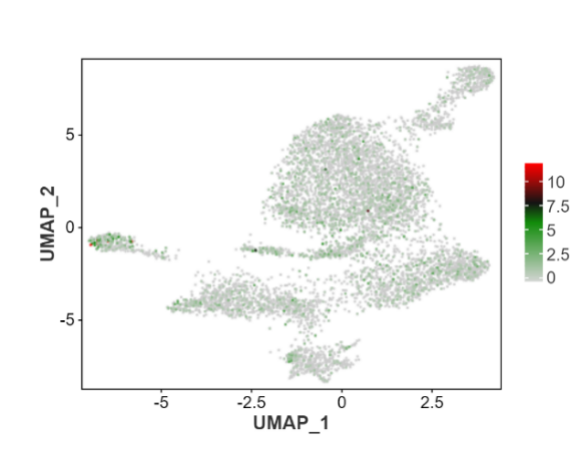


**Gb_05331 (FDH) Gb_15579 (SCRM2)**


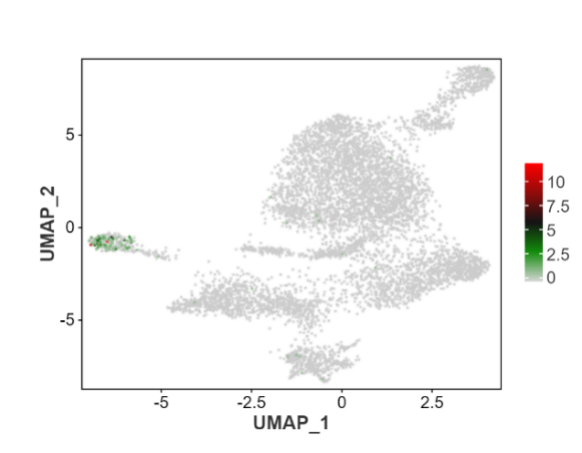

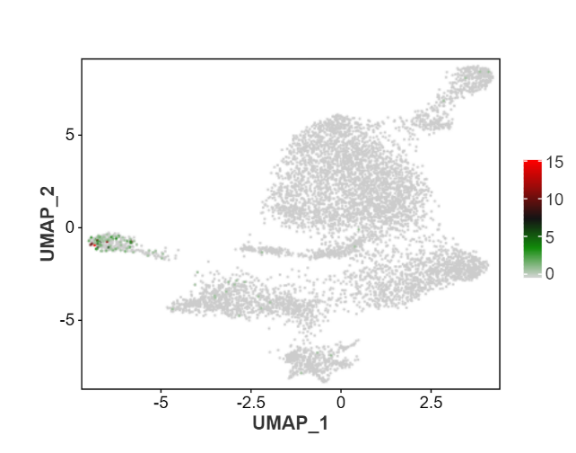


**Gb_32388 (GLIP5) Gb_33524 (HTH)**


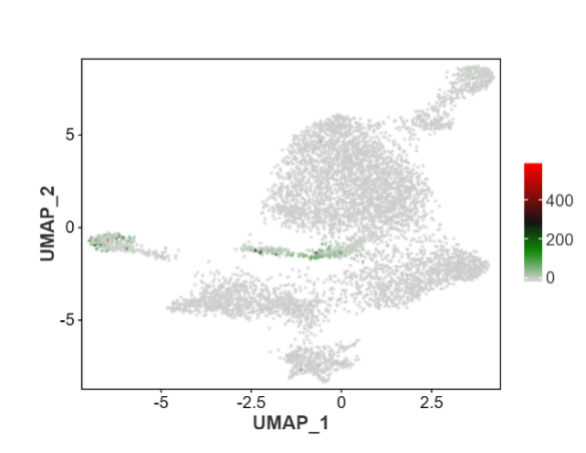


**Gb_41630 (LTP4)**

**Supplementary Figure 19.** **UMAP plot showing the representative marker genes of cellular cluster 6.**

**
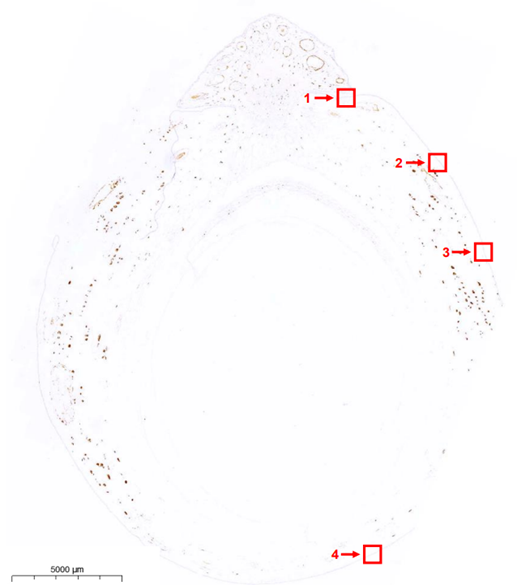
**

**
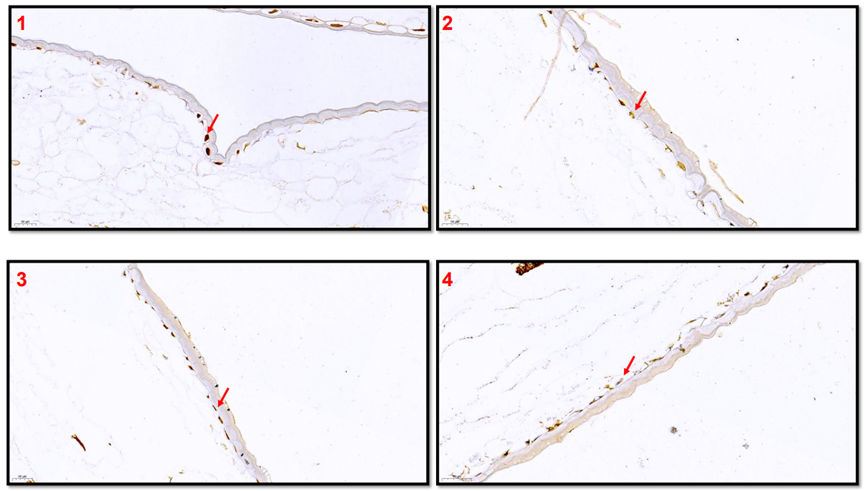
**

**Supplementary Figure 20. RNA *in situ* hybridization of** ***Gb_05331* (*FDH*), which is marker gene for the epidermis cells (ECs).**

* The red arrows indicate cells with successful RNA in situ hybridization. Hematoxylin stains the cell nucleus blue, and DAB shows cell nucleus as brownish-yellow. The darker the brownish-yellow color of the cell nucleus, the higher the expression level.


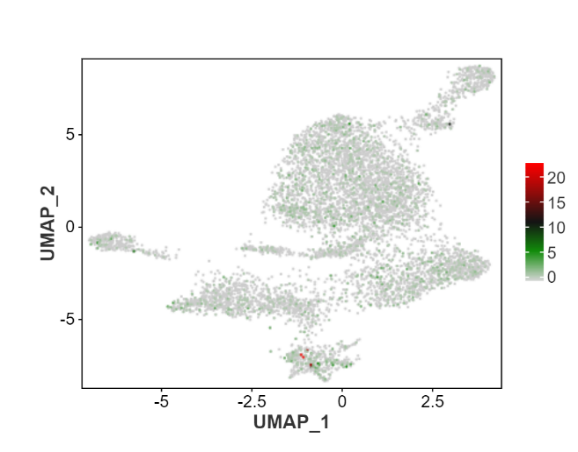

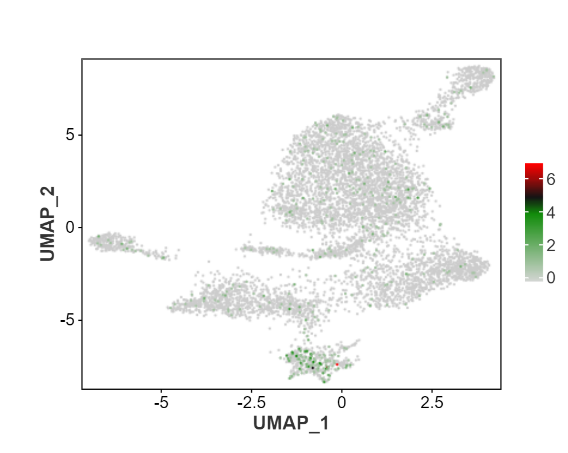


**Gb_05750 (LSD1) Gb_28017 (At3G52120)**

**Supplementary Figure 21. UMAP plot showing the representative marker genes of cellular cluster 5.**

**
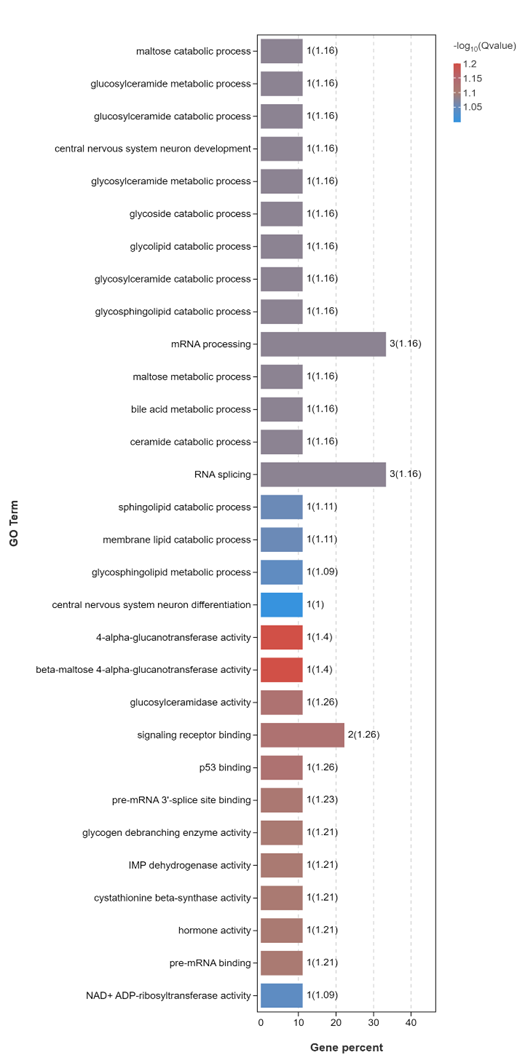
**

**Supplementary Figure 22. Differentially expressed gene ontology (GO) enrichment analysis of up-regulated genes in cellular cluster 5.**

**
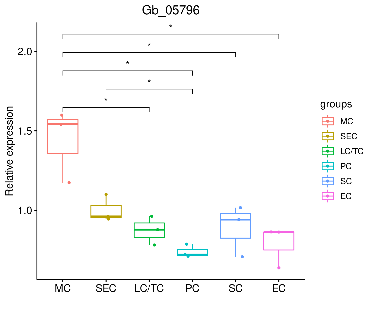

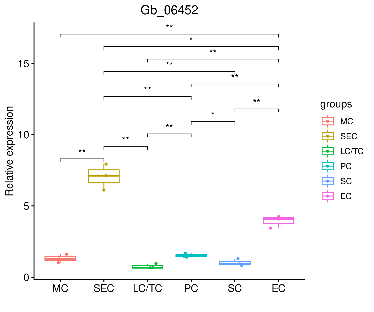

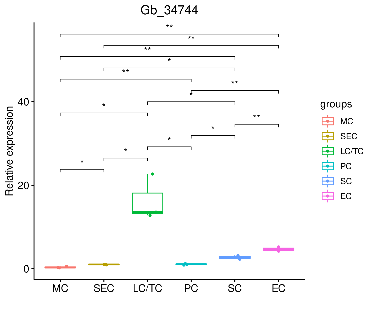
**

**
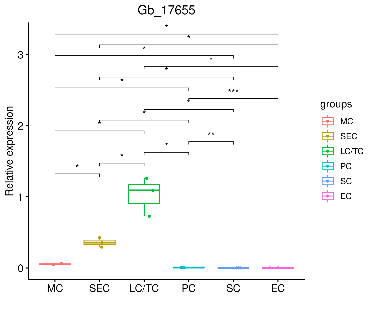

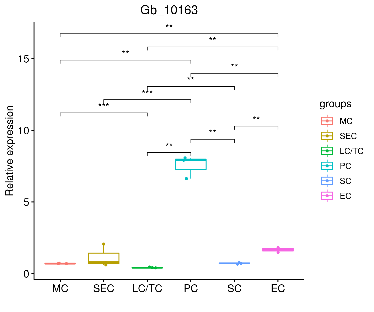

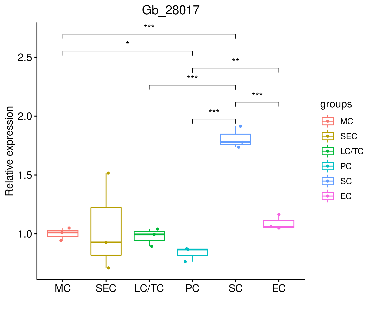
**

**
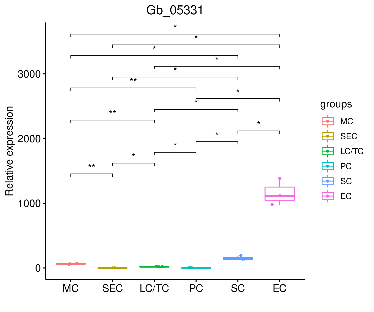
**

**Supplementary Figure 23. Relative expression of marker genes in different cellular clusters.**

* 0.01<p<0.05 as *, 0.001<p<0.01 as **, p<0.001 as ***.

**
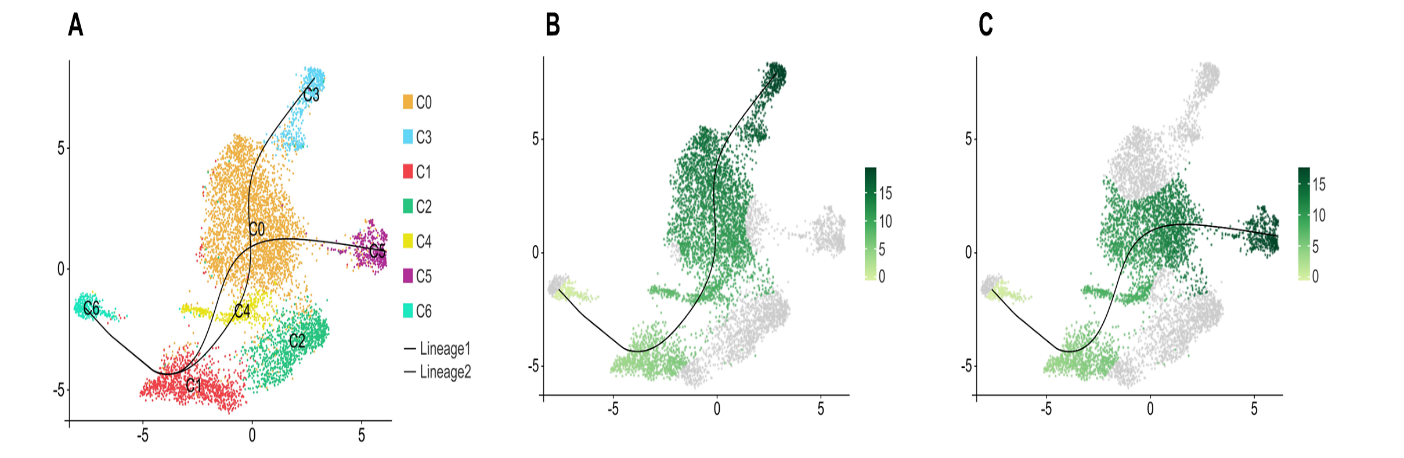
**

**Supplementary Figure 24. Pseudotime analysis of cells with the Slingshot method.** A. The two linages represent two differentiation direction. B. Lineage 1: trajectory of cells differentiating into tracheid cells. C. Lineage 2: trajectory of cells differentiating into secretory cavity cells.

The cellular clusters 0, 1, 2, 3, 4, 5, and 6 are MCs, SECs, LCs, TCs, PCs, SC cells, and ECs. The latent time of the colors in pictures B and C represents different differentiation times, with lighter green indicating earlier times and darker green indicating later times.

**C0**
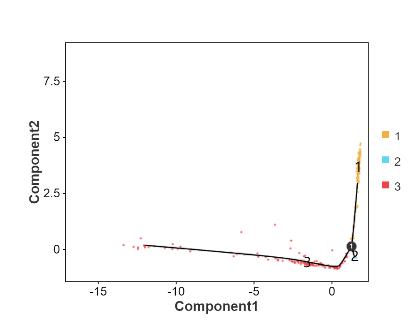

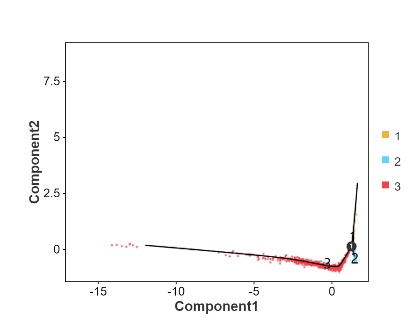


**C1**
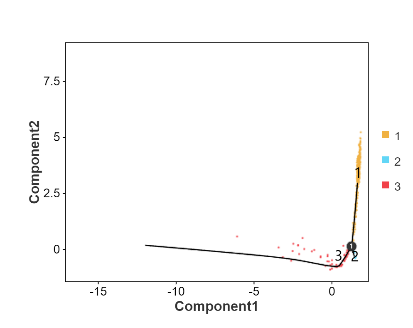

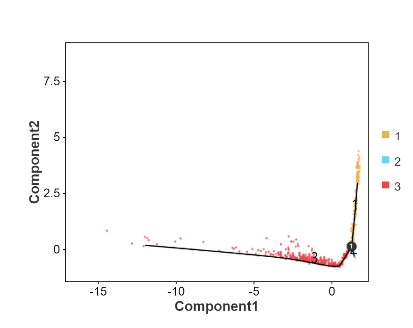


**C2**
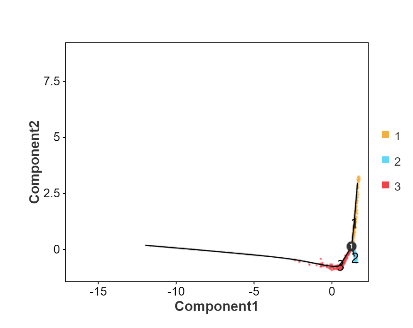

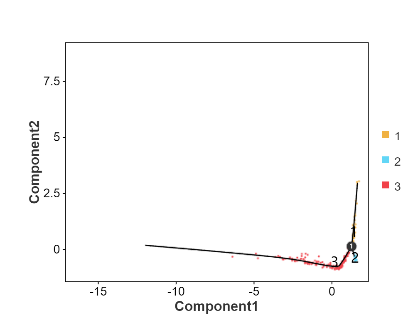


**C3**
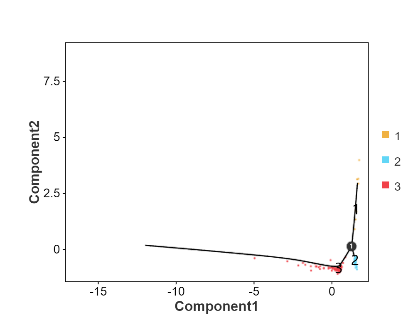

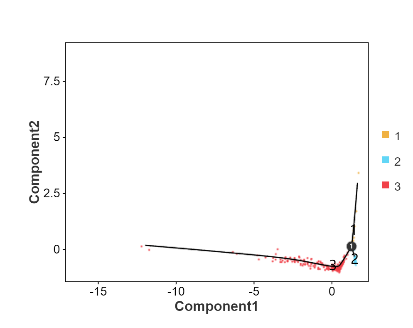


**C4**
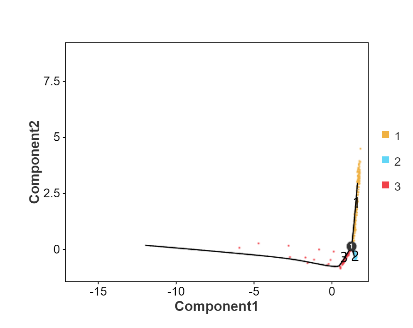

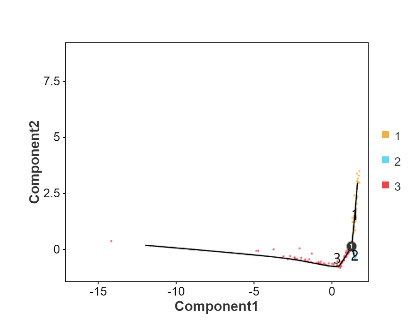


**C5**
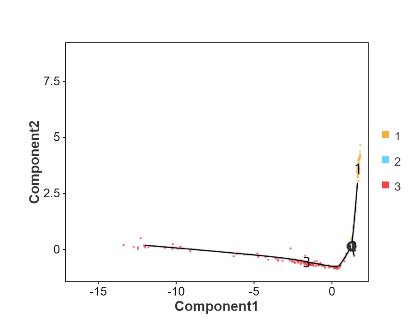

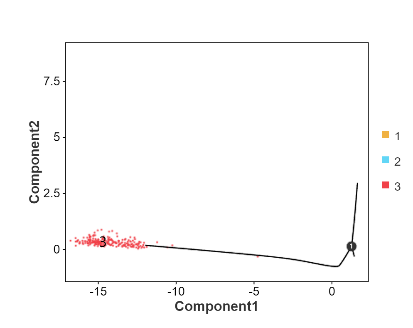


**C6**
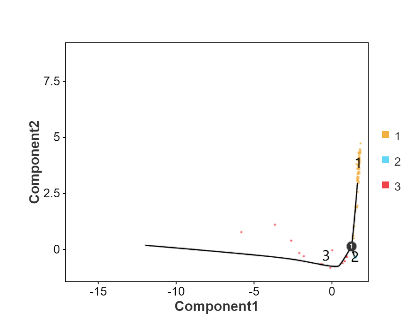

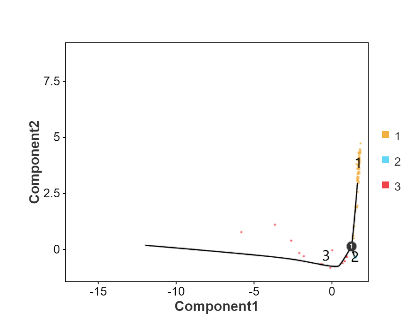


**CK JA**

**Supplementary Figure 25. RNA velocity analysis of six cellular clusters mapped three cellular states in the pseudotime plot.**

**CK1
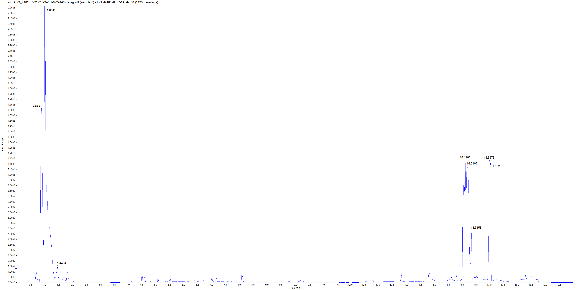

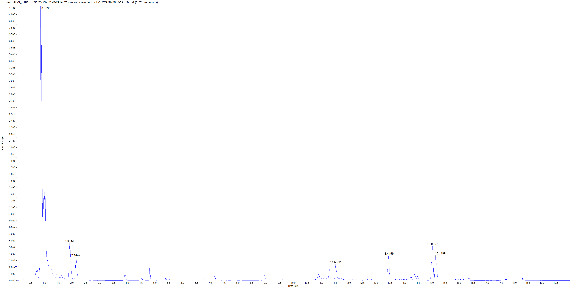
**

**CK2
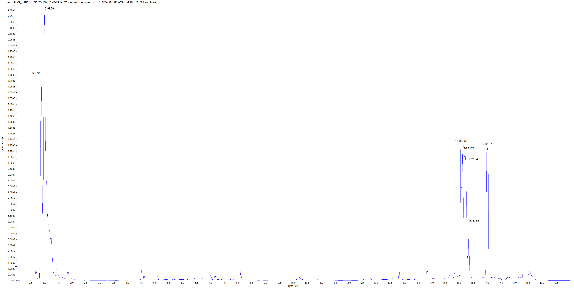

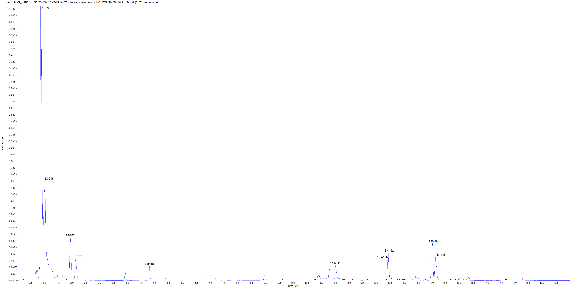
**

**CK3
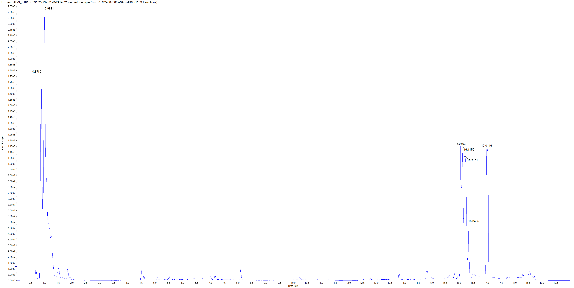

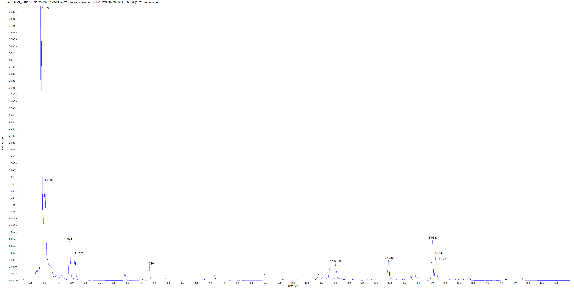
**

**JA1
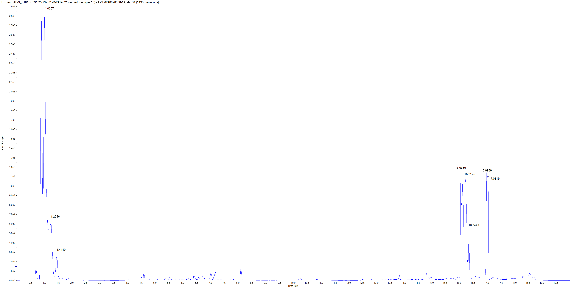

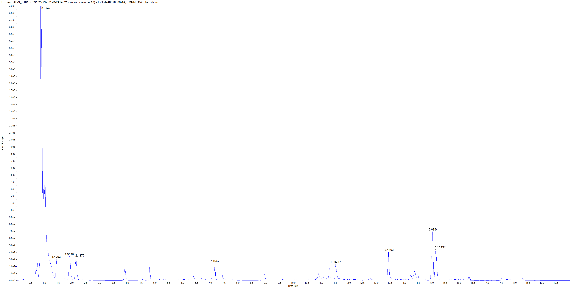
**

**JA2
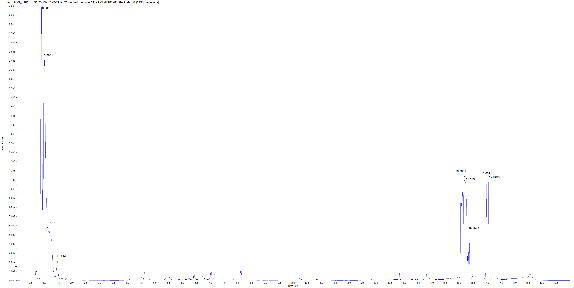
**

**JA3**

**Negative model Positive model**

**Supplementary Figure 26. TIC plot in all samples.**

**Supplementary Figure 27. PCA of Metabolites in CK and JA group.**

**Supplementary Figure 28. OPLS-DA scoring plot.**

**Supplementary Figure 29. Phylogenetic analysis of the AAE gene family of *Arabidopsis thaliana* and *Ginkgo biloba***

**Supplementary Figure 30. Relative expression levels of 14 node genes in 7 cellular clusters**

**Supplementary Figure 31. Molecular structure prediction of GbWRKY35.** A: Secondary structure components of GbWRKY35. B: Prediction of secondary structure of GbWRKY35. C: GbWRKY35 domain prediction.

**Supplementary Figure 32. Prediction of hydrophilicity, transmembrane regions and signal peptides of GbWRKY35 protein.** A: Prediction of hydrophilicity of GbWRKY35 protein. B: Prediction of transmembrane regions of GbWRKY35 protein. C: Prediction of signal peptide of GbWRKY35 protein.

**Supplementary Figure 33. GbWRKY35 (Gb_25334) conserved domain multiple sequence alignment.**

**Supplementary Figure 34. pHIS2-Gb_18621*pro* + pGADT7 self-activation inhibition 3AT concentration screening.**

pHIS2-Gb_18621*pro*: self-activation detection group, pHIS2-p53+pGAD53m: positive control group.

**Supplementary Figure 35. pHIS2-Gb_31209*pro* + pGADT7 self-activation inhibition 3AT concentration screening.**

pHIS2-Gb_31209*pro*: self-activation detection group, pHIS2-p53+pGAD53m: positive control group.

**Supplementary Figure 36. Self-activation detection of pGBKT7-*Gb_25334*+pGADT7.**

**Supplementary Figure 37. pGBKT7-*Gb_25334*+pGADT7 self-activation inhibition 3AT concentration screening.**

**Supplementary Figure 38. Screening of *G. biloba* cDNA library based on yeast two-hybrid system.**

pGBKT7-laminC+pGADT7-largeT: positive control group.

**Supplementary Figure 39. The content distribution of** **hydroxypropylcellulose in *G. biloba* seed.** Different colors represent different relative content, with darker shades of blue indicating lower content and darker shades of red indicating higher content.

**Supplementary Figure 40. The Gene Regulatory Network (GRN) was constructed from DEGs of 51 transcription factor-encoding genes, 2 enzyme-encoding genes in ginkgolic acid biosynthesis and other enzyme-encoding genes.** The size of the node represents the number of gene connections. The color of the node, ranging from green to blue to gray to red, indicates the abundance of gene connectivity from low to high. Circles and diamond represent transcription factor-encoding genes and enzyme-encoding genes, respectively.
